# Roars, Rumbles, and Resonance: A Systematic Review and Meta‐Analysis of Crocodylian Acoustic Signals

**DOI:** 10.1002/ece3.72494

**Published:** 2026-01-22

**Authors:** Sonnie A. Flores, Ross G. Dwyer, Stuart Parsons, Dominique A. Potvin

**Affiliations:** ^1^ School of Science, Technology and Engineering University of the Sunshine Coast Sippy Downs Queensland Australia

**Keywords:** bioacoustics, crocodilian, indices, monitoring, reptile, vocal

## Abstract

Crocodylians are highly vocal reptiles, possessing a complex acoustic signalling system including vocal and non‐vocal signals used for courtship, mating, mediating conflict, and providing maternal care. Despite this, research on crocodylian acoustic signalling remains infrequent, with methodologies and terminology varying widely across studies. Here, we systematically review the literature and standardise crocodylian acoustic signal descriptions, measurements, and terminology to establish a consistent foundation for future research. The compiled dataset included 22 publications, with 623 acoustic signalling observations and 53 distinct parameters measured across various species, life stages, and contexts. The American alligator ( *Alligator mississippiensis* ) was the most frequently studied species and distress calls the most commonly recorded signal type. Significant variation existed in parameters measured across studies, with dominant frequency, call duration, and fundamental frequency the most common. We synthesised acoustic parameters from available publications into comparable values and units, and provide these as a centralised database along with a standardised ethogram including representative spectrograms, and a glossary of commonly used terms facilitating clearer cross‐species comparisons. Significant foundational level data gaps were identified with many species lacking defined repertoires, and notably, underwater acoustic signalling was rarely incorporated. We recommend shifting future research focus from distress calls to include a wider range of spontaneously produced acoustic signals, by individuals of known sex and life stage. The inclusion of a broader set of underrepresented acoustic parameters will also enable better cross‐species comparisons, and identification of encoding in crocodylian acoustic signals. We further promote the reanalysis of existing data incorporating these key parameters, along with increased collaborative efforts, to add valuable insights informing conservation without requiring additional fieldwork. Such strategies will support future research into crocodylian acoustic communication and guide the development of more effective monitoring techniques such as passive acoustic monitoring and machine learning as tools for conservation and management of crocodylians.

## Graphical abstract

Crocodylians are highly vocal reptiles, possessing a complex acoustic signalling system including vocal and non‐vocal signals used for courtship, mating, mediating conflict, and providing maternal care. Despite this, research on crocodylian acoustic signalling remains infrequent, with methodologies and terminology varying widely across studies. Here, we systematically review the literature and standardise crocodylian acoustic signal descriptions, measurements, and terminology to establish a consistent foundation for future research. Photo credit: Mr. David White.

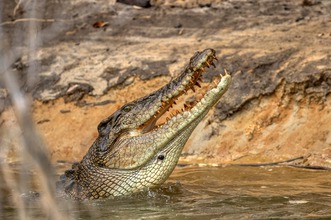

## 1 Introduction

Crocodylians are cryptic apex predators inhabiting terrestrial, fresh and saltwater ecosystems in tropical and sub‐tropical latitudes worldwide (Vergne et al. 2009; Lourenço‐de‐Moraes et al. 2023). Having diverged from a common ancestor around 240 million years ago, crocodylians and birds both sit within the Archosaurian group and share traits associated with vocal communication (Abzhanov 2015; Reber et al. 2017; Chen and Wiens 2020). For example, both vocalise for courtship, mating, and parental care, and show similarities in neocortical cell types, auditory apparatus and unidirectional respiration (Riede et al. 2011; Briscoe et al. 2018). With over half of the 28 species of extant crocodylians listed as threatened by the International Union for the Conservation of Nature (IUCN 2024; Lourenço‐de‐Moraes et al. 2023) and a pervasive global perspective of crocodiles as a threat or conflicting with human interests (Brien et al. 2017), reliable, cost‐effective and non‐invasive monitoring methods such as bioacoustics are becoming increasingly necessary for conservation efforts.

One challenge regarding the study and quantification of crocodylian acoustic signals is their variability: crocodylians produce a wide range of these signals, through various means. These include vocalisations such as growls, roars (in crocodiles) and bellows (in alligators), all of which are produced by air pressure travelling through the laryngeal vocal folds causing them to vibrate through the trachea, with resonance produced by the vocal tract (Riede et al. 2011; Reber et al. 2017). Respiratory signals differ from vocalisations in that they are produced by forceful exhalation without tracheal vibration or resonance. Examples of these signals include bubbles, narial geysering and hissing. A third means of acoustic signalling is percussion, involving sounds created by body–body or body‐object striking, such as jaw pops and headslaps (Vergne et al. 2009; Riede et al. 2011, 2015). Additionally, recent advances in bioacoustic technology have enabled identification of low frequency underwater signals including moos, rumbles, drums, gusts and moans (Staniewicz et al. 2022, 2023). Each acoustic signal type comes with a suite of spectral characteristics that can be detected and analysed via bioacoustics software. Vocalisations, for example, are composed of a fundamental frequency, harmonics (Vergne et al. 2012), and for some species formants (Reber et al. 2015, 2017), all of which potentially modulate in frequency and amplitude across time (Vergne et al. 2012). These modulations can make vocalisations appear indistinct on a spectrogram, thus making crocodylian vocalisations complex to measure. Thus, another major challenge facing crocodylian bioacoustics research is the ability to quantify and compare the seemingly innumerable spectral features that might vary within and between acoustic signals. This complexity can present analytical difficulties, at the same time opening avenues for detailed investigations into behavioural context and signal function.

Acoustic signals not only vary in their structure, but also in the contexts in which they are produced and the information they convey. Overall, crocodylians appear most vocal during the mating season (Garrick and Lang 1977) and at night (Vliet 1989; Brien et al. 2013; Campbell et al. 2014; Staniewicz et al. 2023). As part of a complex multi‐modal communication system, acoustic signalling is thought to structure crocodylian social hierarchies, negotiate courtship and mating, and indicate territoriality (Garrick and Lang 1977; Staniewicz et al. 2022; Walsh et al. 2022). These functions generally require signals to convey honest, individual information regarding traits that are difficult to falsify (Ruxton and Schaefer 2011). In many birds and mammals, formants (i.e., resonance frequency of the vocal tract) serve as honest signal components, as their production is constrained by physiology (Reber et al. 2017). Such formants exist in the bellows produced by the Chinese alligator (*Alligator sinensis*, Fauvel 1879) (Reber et al. 2015) and likely also the American alligator (*Alligator mississippiensis*, Daudin 1801) (Reber et al. 2017). This indicates that formants could be a candidate feature of crocodylian vocalisations that could convey honest and/or individual information (Reber et al. 2015, 2017; Jensen et al. 2024), but this has yet to be tested.

Despite the importance of acoustic signalling to our understanding of crocodylian biology, limited research exists quantifying the acoustic structure, context and signalling behaviour of species worldwide. Crocodylian population monitoring and management is becoming increasingly important, particularly as technological advances have expanded the possibilities for acoustic monitoring making this technology more accessible, scalable and cost‐effective. Coupled with the rise of automated analysis through machine learning—monitoring crocodylians by their acoustic signals is now achievable. However, to fully realise the potential of this important field and associated management approaches, there is an urgent need to systematically review existing studies, resolve inconsistencies in the literature and establish standardised methodologies. Standardising methodologies will avoid confusion and enable meaningful comparisons across species, populations, sexes, developmental stages, and behavioural contexts (Broughton 1963; Ruxton and Schaefer 2011; Kershenbaum et al. 2016). Such issues have been longstanding in the field of bioacoustics in other taxa such as amphibians and birds, whereby multiple terms may be used for the same metric, and single terms have multiple definitions (Köhler et al. 2017; Odom et al. 2021). Such inconsistencies are even found within studies of the same species (Odom et al. 2021). As such, a standardised framework of terminology, behaviours and acoustic signals is advised as a guide for future study into acoustic signalling among crocodylians.

In this paper, we pursue three main aims. First, we systematically review the peer‐reviewed literature on crocodylian vocalisations. Second, we extract and synthesise data from these studies to catalogue the types of acoustic signals described, the parameters measured, and the behaviours associated with each signal. To our knowledge, there is no peer‐reviewed publication that consolidates and standardises acoustic signal measurements collected from crocodylians in captive and wild settings. This is significant as understanding acoustic signal measurements is essential for interpreting species‐specific communication strategies, ecological adaptations and behavioural functions (Alcocer et al. 2022). Our paper synthesises these measurements, allowing for comparison across species, sex, life stage and contexts. Finally, we identify cross‐species and contextual patterns in vocalisations, along with any biases in the existing literature related to sex, life stage, or species representation. These findings will support three key outcomes: (i) recommendations for more effective acoustic monitoring of crocodylian species, (ii) a proposed unified terminology for categorising and describing vocalisations, and (iii) the identification of critical gaps in the existing literature that warrant further investigation. We also explore the broader implications of our findings for the use of passive acoustic monitoring in crocodylians, offering guidance for the development of more advanced monitoring techniques and contributing to a deeper understanding of their acoustic communication.

## 2 Methods

### 2.1 Literature Search and Review

**FIGURE 1:**
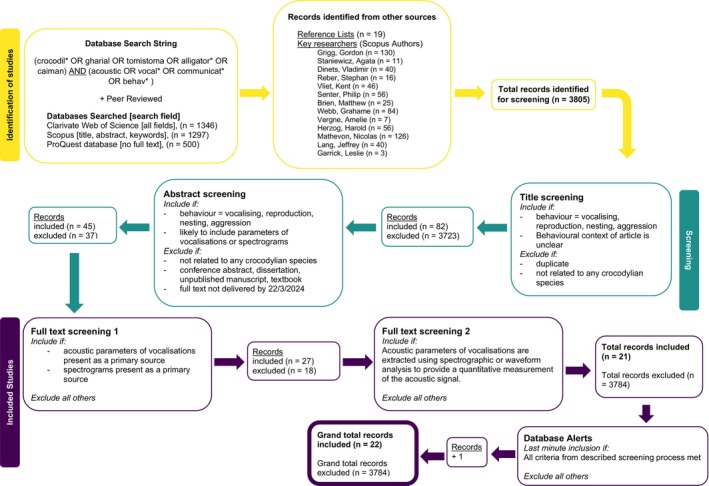
Flowchart showing details of the literature screening process and decision tree, adapted from Page et al. (2021), PRISMA 2020 flow diagram.

We searched the peer‐reviewed literature for studies investigating acoustic signalling in crocodylians up to and including a publication date of 15 February 2024. We used the PICO (population, intervention, components, outcome) framework (Richardson et al. 1995) to define our search string [(crocodil* OR gharial OR tomistoma OR alligator* OR caiman) AND (acoustic OR vocal* OR communicat* OR behav*)] (Figure 1). Searches were performed in Clarivate Web of Science, Scopus, and Proquest databases. Keywords were searched in the ‘title, abstract and keywords’ of the references in Scopus and Proquest, and in ‘all fields’ in Clarivate Web of Science. We also included additional records identified through searches of the reference lists of previously included studies, along with a search of key researchers performed in Scopus Authors on 23 February 2024 (Figure 1).

We conducted a systematic screening process to include only peer‐reviewed, primary‐source articles reporting quantitative acoustic signal parameters of crocodylians extracted from spectrograms. During title screening, we selected studies that explicitly mentioned vocalisations, acoustic signalling, communication, or behaviours related to reproduction, nesting, or aggression. Articles with unclear titles regarding acoustic signalling behaviour were also included to avoid missing studies that may have measured acoustic parameters incidentally. Duplicate entries and articles unrelated to crocodylians were excluded at this stage.

Abstract screening further refined the selection by identifying studies discussing crocodylian (vocal and non‐vocal) acoustic signals, reproduction, nesting, or aggression, or those likely to contain acoustic measurements or spectrograms. Unpublished manuscripts, textbooks, conference abstracts, and dissertations were excluded to ensure the inclusion of peer‐reviewed publications only. Alerts from Web of Science and Scopus were set to track new publications meeting this review criteria. As a result, a highly relevant late‐stage publication (Ajji and Lang 2025) was identified and included due to its essential contribution to this body of work. Full‐text screening involved two stages. In the first stage, articles were assessed to confirm the inclusion of acoustic signal parameters or spectrograms as primary sources, excluding duplicates where data appeared in review articles but originated from primary studies. In the second stage, only articles that provided quantitative acoustic measurements derived from spectrograms were retained. This approach ensured that all included data was reliable, repeatable, and suitable for synthesis.

### 2.2 Data Extraction and Synthesis

We extracted the following components measured within each article: acoustic signal type (e.g., ‘growl’), parameter (e.g., fundamental frequency, Hz), sample location, number of acoustic signals, number of individuals, species, sex, life stage and/or total length, and context. We excluded measurements related to sound pressure level and harmonics from this review as these measurements are often influenced by environmental noise, recording distances, and other technical constraints. Similarly, we excluded physiological parameters such as subglottal pressure from our analysis since they are not observable by passive acoustic monitoring or in field studies without invasive procedures.

Common terms for specific signal types were chosen to represent the same functionally equivalent signal, e.g., ‘roars’ (Crocodylidae) and ‘bellows’ (Alligatoridae), which were combined under the term ‘bellows’ (Grigg and Kirshner 2015; Jensen et al. 2024). Upon examination of the spectrograms, the signal, referred to as a ‘Moo’ by both Staniewicz et al. (2023) and Wang et al. (2007) appeared to exhibit distinct spectrographic characteristics, suggesting it may represent acoustically different call types. Therefore, we assigned a numerical designation in our dataset to the ‘Moo’ reported by Staniewicz et al. (2023) (designated ‘Moo‐1’) to distinguish these calls from one another. Original call names and categorisations are detailed in Supporting Information Table S4 and listed as alternative terms in the ethogram (Table 3).

Where possible, the acoustic signal parameters for each call type were summarised into comparable mean ± standard deviation (SD) values, with units converted into a common unit (e.g., kHz converted to Hz). Unit conversions and calculation of standardised mean ± SD values were performed in Microsoft Excel (version 1208, Build 14334.20244). Detail of how each acoustic measurement was calculated is provided in Supporting Information Table S4, in column N titled ‘How data was synthesised for this table’, with blank rows indicating no calculation or unit conversion was either necessary or possible for that measurement. Specific formulas for each calculation are provided in Supporting Information Table S5. Life stages were either stated or estimated based on the focal individual's total length (TL) as described in Webb and Messel (1978) (Supporting Information Tables S4 and S5). Parameters that were described using diverse language but that signified a specific, common measurement were consolidated into a grouping using a single, collective term. For example, we use ‘F0 (Hz)’ to represent the mean measurement of the lowest frequency component in a vocal signal. This same parameter is termed ‘mean pitch’ in Chabert et al. (2015); ‘fundamental frequency’ in Reber et al. (2017) and both ‘fundamental frequency’ and ‘pitch’ in Staniewicz et al. (2022). The term ‘F0 (Hz)’ is widely recognised in bioacoustics as fundamental frequency, and thus allows here a unified comparison of measurements describing the same parameter. We have also included alternative terms in our compiled, comprehensive glossary (Supporting Information Table S6). Where the mean could not be calculated for a measurement and a range was provided, we used the lowest value of the range to perform the analysis (e.g., *n* = 20–30 individuals was amended to *n* = 20). This single value was used for our analysis in R (R Core Team 2024) to describe the dataset and create visualisations only (Supporting Information S1 and S2), while range details are retained in the summary table of results (Supporting Information Table S4).

Acoustic parameters were allocated a broad category based on whether they described the frequency energy distribution (FED), time window (TW) or temporal energy distribution (TED) of the acoustic signal measured. Together, these categories have been suggested to illustrate the structural diversity of acoustic signals (Odom et al. 2021). Acoustic parameters that could not be designated to one of these categories were categorised as ‘other’ (OT).

All published acoustic signals and their associated behaviours were categorised through the development of an ethogram. Each published observation was assigned a behavioural context and a method for inducing production of the acoustic signal (if any). Similarly, to investigate how measurements of acoustic signals compared across studies and to examine the dataset for bias, each observation was allocated to a series of categories (i.e., species, sex, life stage, context and method of induction). We then calculated the percentage of observations for each acoustic signal within each category examined. As some papers measured more than one species, life stage, or sex, these categories were applied to ensure the correct number of observations was included therein, for accurate calculation of percentages and counts.

We were unable to perform significance testing on our dataset due to the low degree of consistency in the use of acoustic parameters and the lack of comparable measurements across medium, species, sex, life stage and context. Instead, boxplots were generated to visually assess and compare mean values from data sets across these parameters, which facilitated identification of differences between central tendencies and variability across datasets. No data imputation, statistical corrections or transformations were applied during analysis, as the dataset was complete and free of missing values. Statistical analysis and data visualisation were performed in R (R Core Team 2024).

## 3 Results

### 3.1 Literature Review

A total of 22 publications were included for analysis in our systematic literature review, with the first study published in 1973 (Campbell 1973) and 77% (*n* = 17) published after 2007. The resulting compiled dataset contained 623 distinct observations of acoustic signalling across 53 unique acoustic parameters. The number of acoustic signals collected and measured in each study ranged from 1 to 590, with 280 observations across 5 studies for which the number of acoustic signals measured was either estimated or not provided by the study authors. The number of individuals sampled in each study ranged from 1 to 31 crocodiles of known identity and included groups of crocodiles ranging between 20 and 120 individuals, as well as 67 instances where the exact number of individuals was unknown. Therefore, a large proportion of the data set compiled for this review included observations where the number of calls measured and individuals sampled was estimated or explicitly unknown.

**FIGURE A1:**
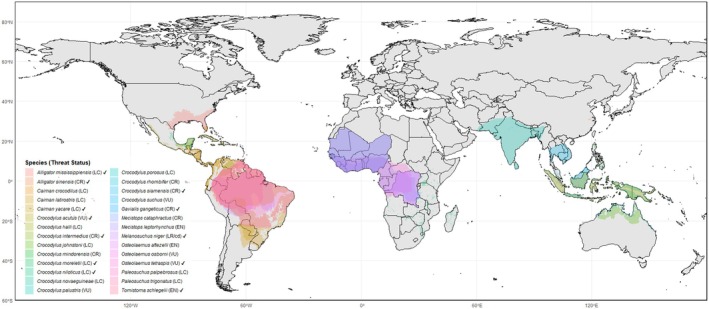
Global map showing the distribution of all 28 extant crocodylian species worldwide. The legend shows the species name with threat status following in brackets, and a tick for species that have acoustic measurements published in the peer‐reviewed literature and are thus included in this review. Map generated in R (R Core Team 2024) using the R package: Rnaturalearth (South et al. 2024) and spatial data from the IUCN (2025). Reference list of all sources used to create Figure A1 are listed below.

Of the 623 distinct observations provided in this review, only 13 of 28 extant crocodylian species were represented. The species measured most frequently was the American alligator with 117 acoustic measurements within the dataset (18.8%; *n* = 117). This was followed by the Nile crocodile ( *Crocodylus niloticus* , Laurenti 1768) and the African dwarf crocodile ( *Osteolaemus tetraspis* , Cope 1861) with 110 and 92 acoustic measurements respectively (17.7%, *n* = 110; 14.8%, *n* = 92). Over half of the extant crocodylian species lacked measurements of any acoustic signals in the published literature (53.6%, *n* = 15) (Table 1), with 53.3% (*n* = 8) of these species listed as threatened (VU, EN, CR) by the IUCN red list (IUCN 2024) (Figure A1).

**TABLE 1:** Species lacking published acoustic measurements along with their geographic distribution and global IUCN red list status.

Species name	Common name	Geographic distribution	Global IUCN red list status	Last assessed	Citation
*Caiman latirostris*	Broad‐snouted caiman	Argentina; Bolivia, Plurinational States of; Brazil; Paraguay; Uruguay	LC	2019	Siroski et al. (2020)
*Crocodylus halli*	Hall's New Guinea crocodile	Indonesia (Papua); Papua New Guinea	LC	Not available	Lourenço‐de‐Moraes et al. (2023); Murray et al. (2024)
*Crocodylus johnstoni*	Australian freshwater crocodile	Australia	LC	2016	Isberg et al. (2017)
*Crocodylus mindorensis*	Phillipine crocodile	Philippines	CR	2012	van Weerd et al. (2016)
*Crocodylus novaeguineae*	New Guinea freshwater crocodile	Indonesia (Papua); Papua New Guinea	LC	2018	Solmu and Manolis (2019)
*Crocodylus palustris*	Mugger crocodile	India; Iran, Islamic Republic of; Nepal; Pakistan; Sri Lanka	VU	2009	Choudhury and de Silva (2013)
*Crocodylus porosus*	Saltwater/estuarine crocodile	Australia; Bangladesh; Brunei Darussalam; India; Indonesia; Malaysia; Myanmar; Palau; Papua New Guinea; Philippines; Singapore; Solomon Islands; Sri Lanka; Timor‐Leste; Vanuatu	LC	2019	Webb et al. (2021)
*Crocodylus rhombifer*	Cuban crocodile	Cuba	CR	2022	McMahan et al. (2022)
*Crocodylus suchus*	West African crocodile	West African nations	VU	not available	Hekkala et al. (2011); Lourenço‐de‐Moraes et al. (2023)
*Mecistops cataphractus*	African slender‐snouted crocodile	Cameroon; Central African Republic; Congo; Congo, The Democratic Republic of the; Côte d'Ivoire; Gabon; Gambia; Ghana; Liberia; Sierra Leone	CR	2013	Shirley (2014)
*Mecistops leptorhynchus*	Central African slender‐snouted crocodile	West‐central African nations	EN	Not available	Lourenço‐de‐Moraes et al. (2023)
*Osteolaemus aftezelli*	West African dwarf crocodile	West‐central African nations	EN	Not available	Lourenço‐de‐Moraes et al. (2023)
*Osteolaemus osborni*	Osborns's dwarf crocodile	West African nations	VU	Not available	Lourenço‐de‐Moraes et al. (2023); Smolensky et al. (2015)
*Paleosuchus palpebrosus*	Cuvier's dwarf caiman	Bolivia, Plurinational States of; Brazil; Colombia; Ecuador; French Guiana; Guyana; Paraguay; Peru; Suriname; Trinidad and Tobago; Venezuela, Bolivarian Republic of	LC	2018	Magnusson et al. (2019)
*Paleosuchus trigonatus*	Schneider's smooth‐fronted caiman	Bolivia, Plurinational States of; Brazil; Colombia; Ecuador; French Guiana; Guyana; Peru; Suriname; Venezuela, Bolivarian Republic of	LC	2018	Campos et al. (2019)

Abbreviations: CR, CriticallyEendangered; EN, Endangered; LC, Least Concern; VU, Vulnerable.

Of these 15 species, five are from Australasia (i.e., Australia and Southeast Asia: *Crocodylus halli, Crocodylus johnstoni, Crocodylus mindorensis, Crocodylus novaeguineae and Crocodylus porosus*), five are from West or West‐Central Africa (*Crocodylus suchus, Mecistops cataphractus, Mecistops leptorhynchus, Osteolaemus aftezelli, Osteolaemus osborni)*, four are from South/Central America (*Caiman latirostris, Crocodylus rhombifer, Paleosuchus palpebrosus, Paleosuchus trigonatus*), and one is from South Asia (i.e., Indian subcontinent: *Crocodylus palustris* ). Those species with acoustic parameters published were also globally distributed, with seven from the Americas, two each from Africa and Southeast Asia, and one each from China and the Indian subcontinent. Six of these are Least Concern, three are Vulnerable and four are listed as Critically Endangered by the IUCN (IUCN 2024) (Figure A1).

### 3.2 Acoustic Signals, Associated Behaviours and Parameter Measurements

We found a total of 19 distinct acoustic signal types described across 13 crocodylian species (Table 2).

**TABLE 2:** Call types included in this review detailing the species, publication, sex and life stage for each.

Call type	Species	Citation	Sex			Life stage					
			Female	Male	Unknown	Adult	Hatchling	Juvenile	Pre‐hatching	Sub‐adult	Unknown
Bellow	*Alligator mississippiensis*	Garrick et al. (1978) Reber et al. (2017) Todd (2007) Vliet (1989)	X	X	X	X					
	*Alligator sinensis*	Reber et al. (2015) Wang et al. (2007)	X		X	X					
	*Melanosuchus niger*	Vergne et al. (2009)	X			X					
Bubbles	*Tomistoma schlegelii*	Staniewicz et al. (2022)			X	X					
Contact call	*Alligator mississippiensis*	Herzog and Burghardt (1977) Riede et al. (2011)			X			X			
	*Caiman crocodilus*	Campbell (1973) Vergne et al. (2012)			X		X	X			
	*Crocodylus acutus*	Campbell (1973)			X				X		
	*Crocodylus niloticus*	Vergne et al. (2009) Vergne et al. (2012)			X		X		X		
	*Melanosuchus niger*	Vergne et al. (2011) Vergne et al. (2012)			X		X	X			
Cough	*Alligator mississippiensis*	Garrick et al. (1978)			X	X					
	*Tomistoma schlegelii*	Staniewicz et al. (2022)			X	X					
Distress call	*Alligator mississippiensis*	Chabert et al. (2015) Herzog and Burghardt (1977)			X		X	X		X	
	*Caiman crocodilus*	Chabert et al. (2015) Garrick and Garrick (1978) Herzog and Burghardt (1977) Roberto and Botero‐Arias (2013)			X		X	X			
	*Caiman yacare*	Sicuro et al. (2013)			X		X				
	*Crocodylus acutus*	Boucher et al. (2020) Campbell (1973)			X		X	X			
	*Crocodylus intermedius*	Chabert et al. (2015)			X		X	X		X	
	*Crocodylus moreletii*	Chabert et al. (2015)			X			X			
	*Crocodylus niloticus*	Chabert et al. (2015) Herzog and Burghardt (1977) Vergne et al. (2007) Vergne et al. (2009)			X		X	X		X	
	*Crocodylus siamensis*	Herzog and Burghardt (1977)			X			X			
	*Gavialis gangeticus*	Bonke et al. (2015)			X			X			
	*Melanosuchus niger*	Vergne et al. (2011)			X			X			
	*Tomistoma schlegelii*	Bonke et al. (2015)			X			X		X	
Drum	*Osteolaemus tetraspis*	Staniewicz et al. (2022)			X	X					X
	*Tomistoma schlegelii*	Staniewicz et al. (2022)			X	X					
Growl	*Alligator mississippiensis*	Garrick et al. (1978)			X	X					
	*Crocodylus acutus*	Campbell (1973)			X			X			
Grunt	*Alligator mississippiensis*	Garrick et al. (1978)			X	X					
	*Caiman crocodilus*	Vergne et al. (2009)	X			X					
Gust	*Osteolaemus tetraspis*	Staniewicz et al. (2023)			X	X					X
Headslap	*Alligator mississippiensis*	Garrick et al. (1978), Vliet (1989)			X	X					
Hiss	*Alligator mississippiensis*	Garrick et al. (1978)			X	X					
	*Alligator sinensis*	Wang et al. (2007)			X	X					
	*Caiman yacare*	Sicuro et al. (2013)	X			X					
Moan	*Tomistoma schlegelii*	Staniewicz et al. (2022)			X	X					
Moo	*Alligator sinensis*	Wang et al. (2007)			X	X					
Moo‐1	*Osteolaemus tetraspis*	Staniewicz et al. (2023)			X	X					X
Pop	*Gavialis gangeticus*	Ajji and Lang (2025)		X		X					
Rumble	*Osteolaemus tetraspis*	Staniewicz et al. (2023)			X	X					X
	*Tomistoma schlegelii*	Staniewicz et al. (2022)			X	X					
Sub‐audible vibration	*Alligator mississippiensis*	Vliet (1989)		X		X					
Toot	*Alligator sinensis*	Wang et al. (2007)			X	X					
Whine	*Alligator sinensis*	Wang et al. (2007)			X	X					

The distress call was the most represented call type comprising 58.2% (*n* = 362) of all calls, followed by the contact call which comprised just 9.5% (*n* = 59) (Figure 3). The distress call was represented most broadly across crocodylian species (84.6%, *n* = 11) with only the Chinese alligator and African dwarf crocodile missing measurements for this call type (Figure 3a). Based on the synthesis of reviewed studies (Supporting Information Table S4), we compiled an ethogram of crocodylian acoustic signals (Table 3). This ethogram includes signal names, associated behavioural contexts, observed species, and representative spectrograms. Where relevant, alternate names from the literature were noted. Behavioural postures, qualitative sound descriptions, and key acoustic parameters were summarised.

**TABLE 3:** Ethogram of crocodylian acoustic signals, including signal names, contexts, species observations, and a representative spectrogram.

Acoustic signal [Alternate terms]	Species observed *measured	Description		Example spectrogram/Waveform	Spectrogram/Waveform details
Bellow (Alligatoridae)[Roar (Crocodylidae)]	*A. mississippiensis* *^16^ ^,^ ^17^ ^,^ ^18^ ^,^ ^23^ ^,^ ^24^ ^,^ ^34^ ^,^ ^39^ ^,^ ^40^ *A. sinensis* *^22^ ^,^ ^41^ *C. intermedius* ^33^ *C. mindorensis* ^26^ *C. niloticus* ^20^ *G. gangeticus* ^1^ *M. niger* *^36^	Demographics	Adult male and female	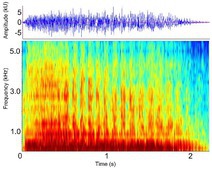	Species: *A. mississippiensis* ## (Jensen et al. 2024) Sample rate (Hz): 44,100 Equipment: Microphone Overlap: 50% Brightness: 50% Contrast: 50%
		Acoustic origin	Vocal tract resonance^22^ ^,^ ^23^, subglottal pressure^23^, and vocal fold adduction^24^		
		Production posture/movement	Produced in air with the head at an oblique angle to the surface of the water, with the tail arched. The vocalising animal inhales, expanding the gular pouch, opens the jaws and emits the vocalisation. The gular glands may be everted. The back of the animal may become exposed above the water surface once the vocalisation begins and small water droplets/vibrating waves may be seen across the back^14^ ^,^ ^16^ ^,^ ^39^		
		Description of signal	Deep, throaty, sonorous^14^ ^,^ ^16^ ^,^ ^39^		
		Emission context/potential function	Advertisement, aggression, territoriality, courtship^14^ ^,^ ^17^/Individual identity, sex identity, fitness^22^ ^,^ ^23^		
		Some defining spectral and temporal characteristics	May be repeated by the same individual^17^ ^,^ ^39^ May be produced in a chorus of multiple individuals^17^ ^,^ ^39^ Harmonics present Formants present ( *A. mississippiensis* ^23^, *A. sinensis* ^22^) Energy concentrated in the lower frequencies Dominant frequency (Hz): 35.5–250.0^17^ ^,^ ^22^ ^,^ ^23^ ^,^ ^34^ ^,^ ^39^ F0 (Hz): 54–56^23^ Duration (s): 0.43–1.83^17^ ^,^ ^22^ ^,^ ^36^ ^,^ ^41^		
Bubbles [Bubble blow, bubbling]	*A. mississippiensis* ^5^, ^16^, ^17^, ^18^, ^19^, ^39^ *A. sinensis* ^41^ *C. intermedius* ^33^ *C. johnstoni* ^10^ *C. mindorensis* ^26^ *C. niloticus* ^20^ *G. gangeticus* ^1^ *T. schlegelii* *^29^	Demographics	Adult male and female^1^, ^5^, ^10^, ^16^, ^17^, ^18^, ^19^, ^20^, ^26^, ^29^, ^33^, ^39^, ^41^	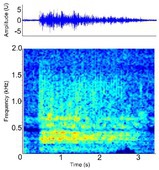	**Species:** *T. schlegelii* ^29^, ## **Sample rate (Hz):** 4000 **Equipment:** Hydrophone **Overlap:** 50% **Brightness:** 50% **Contrast:** 50% **Note:** light blue bands in the spectrogram between the yellow signal represent post‐processing filtering of background noise and do not signify the presence of harmonics.
		Acoustic origin	Nose or mouth^17^ ^,^ ^41^		
		Production posture/movement	Produced underwater by forceful exhalation from the nose or mouth with the head under the surface of the water^29^ ^,^ ^39^ ^,^ ^41^		
		Description of signal	Hollow gurgling		
		Emission context/potential function	Courtship^17^ ^,^ ^29^		
		Some defining spectral & temporal characteristics	Low energy acoustic signal with energy concentrated in the lower frequencies and bandwidth decreasing over time Harmonics absent^17^ Formants unknown Bandwidth (Hz)^W^: 1068 ± 562^17^ Dominant Frequency (Hz)^W^: 407 ± 154^17^ Duration (s)^W^: 2.06 ± 1.54^17^		
Contact call [Bark, chirp, grunt, soft grunt, snort]	A. mississippiensis* ^2^, ^12^, ^16^, ^17^, ^18^, ^19^, ^39^ Ca. crocodilus* ^7^, ^36^, ^38^ C. acutus* ^7^, ^16^ *C. intermedius* ^33^ C. niloticus* ^9^, ^16^, ^31^, ^32^, ^35^, ^36^, ^38^ *G. gangeticus* ^1^ M. niger* ^7^, ^37^, ^38^	Demographics	Pre‐hatching^36^, hatchling^36^ ^,^ ^38^ & juveniles^7^, ^18^, ^24^, ^37^, unknown sex (likely both)	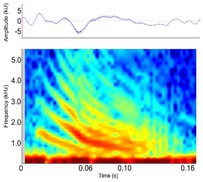	Species: *C. acutus* ^4^, ## Sample rate (Hz): 44,100 Equipment: Microphone Overlap: 50% Brightness: 50% Contrast: 50%
		Acoustic origin	Vocal tract, subglottal pressure, and vocal fold adduction^24^		
		Production posture/movement	Various, but most often with the snout and head raised slightly May be locomoting during vocalisation		
		Description of signal	Short, often repeated chirp‐like sound^7^, ^15^, ^36^		
		Emission context/potential function	Social cohesion, alert to environmental features (i.e., food), individual localisation, interest^7^, ^18^, ^35^, ^36^, ^37^, ^38^		
		Some defining spectral & temporal characteristics	Complex frequency modulation with rapidly decreasing slope and bandwidth^35^ ^,^ ^36^ ^,^ ^37^ ^,^ ^38^ Harmonics present^35^ Formants unknown Bandwidth (Hz): 2573–4996^36^ ^,^ ^39^ Dominant frequency (Hz): 466–1138^36^ ^,^ ^38^ F0 [MAX] (Hz): 292–622^24^ ^,^ ^36^ ^,^ ^37^ ^,^ ^38^ Duration (s): 0.08–0.20^7^, ^24^, ^36^, ^37^, ^38^		
Cough [Chumpf]	A. mississippiensis* ^16^ ^,^ ^17^ ^,^ ^40^ *A. sinensis* ^6^ *C. acutus* ^16^ *C. mindorensis* ^26^ *C. niloticus* ^16^ T. schlegelii* ^29^	Demographics	Adult male & female^17^	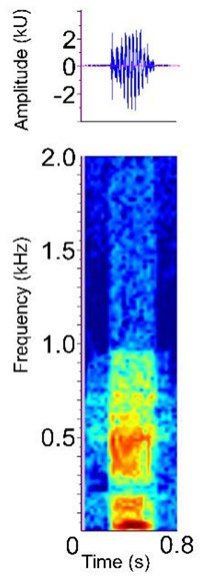	Species: *T. schlegelii* ^29^, ## Sample rate (Hz): 4000 Equipment: Hydrophone Overlap: 50% Brightness: 50% Contrast: 50%
		Acoustic origin	Not specified (likely vocal tract)		
		Production posture/movement	Not specified		
		Description of signal	Deep, short, bark‐like, with abrupt ending^17^ ^,^ ^29^		
		Emission context/potential function	Courtship^17^		
		Some defining spectral & temporal characteristics	Often produced in a sequence of three.^17^ ^,^ ^29^ Detectable both in air and underwater^17^ ^,^ ^29^ Noisy, broadband sound of short duration with energy concentrated in lower frequencies^17^ Harmonics absent^17^ Formants unknown Dominant frequency (Hz)^:^300^17^ Dominant frequency (Hz)^W^: 89 ± 123^29^ F0 (Hz)^W^: 26 ± 12^29^ Duration (s): 0.20–0.31^17^ ^,^ ^29^		
Distress call [Distress grunt, moan (palatal valve closed), screech (palatal valve open), snarl]	A. mississippiensis* ^7^, ^8^, ^16^, ^18^, ^39^ Ca. crocodilus* ^7^, ^8^, ^15^, ^25^ *Ca. yacare* ^28^ *C. acutus* ^4^, ^7^, ^16^ *C. cataphractus* ^27^ *C. johnstoni* ^27^ C. intermedius* ^8^ C. moreletii* ^8^ C. niloticus* ^8^, ^17^, ^35^, ^36^ *C. novaeguineae* ^27^ *C. palustris* ^27^ *C. porosus* ^27^ *C. rhombifer* ^27^ *C. siamensis* ^27^ G. gangeticus* ^3^ *M. niger* ^37^ *O. tetraspis* ^27^ *P. palpebrosus* ^27^ *P. trigonatus* ^27^ T. schlegelii* ^3^	Demographics	All life stages, unknown sex (likely both)	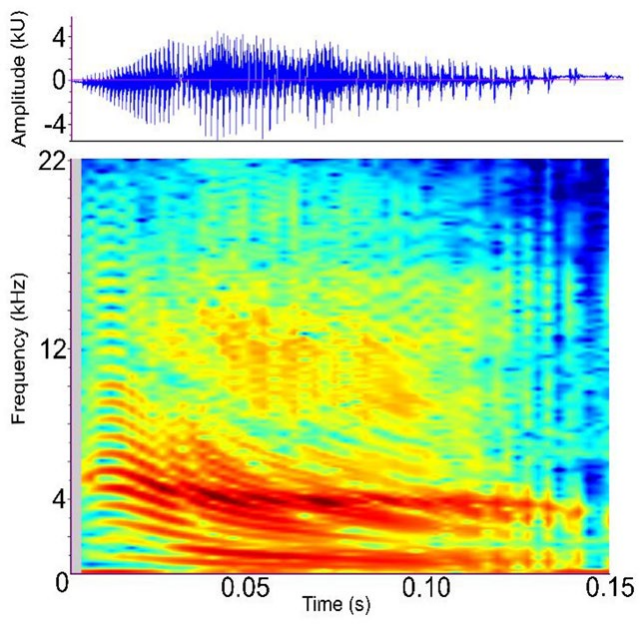	Species: *C. acutus* ^4^, ## Sample rate (Hz): 44,100 Equipment: Microphone Overlap: 50% Brightness: 50% Contrast: 50%
		Acoustic origin	Vocal tract^25^		
		Production posture/movement	Produced in air, with the mouth either open or closed, in a range of postures but most often with the snout and head raised. May be simultaneously locomoting		
		Description of signal	Graded upwards in intensity from the contact call, so is louder, repeated more frequently and has higher amplitude, energy and volume^37^. Varies in frequency depending on the position of the palatal valve (moan‐like = closed, screech‐like = open)^25^ and the life stage of the individual^4^		
		Emission context/potential function	Solicit protection, distress, threat response^3^, ^4^, ^7^, ^8^, ^15^, ^16^, ^17^, ^18^, ^25^, ^35^, ^36^, ^37^		
		Some defining spectral & temporal characteristics	*Hatchlings & juveniles*: complex frequency modulation, higher frequency, shorter duration^4^, ^8^, ^25^, ^35^, ^36^, ^37^ *Sub‐adults & adults*: very little frequency modulation, lower frequency, longer duration^4^, ^8^ Decrease in frequency over call, with steep slope at the end of the call May begin with an initial steeply sloped upsweep Properties vary depending on context^4^, ^28^ Harmonics present^37^ Formants unknown Broad bandwidth up to 15,000 Hz^3^, ^36^ Dominant frequency (Hz): 321 to > 8000^3^, ^18^, ^25^, ^28^, ^36^ F0 (Hz): 222.0–818.0^7^, ^8^ Duration (s): 0.30–1.13^3^, ^4^, ^16^, ^18^, ^25^, ^28^, ^35^, ^36^, ^37^ Peak frequency [Peak 1] (Hz): 3000 ± 1.49		
Drum	*O. tetraspis* ^30^ *T. schlegelii* ^29^	Demographics	Adult, sex unknown (paired male & female observed)^29^ ^,^ ^30^	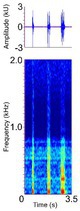	Species: *T. schlegelii* ^29^, ## Sample rate (Hz): 4000 Equipment: Hydrophone Overlap: 50% Brightness: 50% Contrast: 50%
		Acoustic origin	Not specified		
		Production posture/movement	Not specified, measured both in air and underwater & primarily produced at night30		
		Description of signal	Low frequency hollow thump sound that is repeated depending on whether the signal is a single, double or triple drum		
		Emission context/potential function	Not specified^30^, Courtship^s^		
		Some defining spectral & temporal characteristics	Mostly produced singularly but may occur in sequences of up to five or with other signal types^29^ ^,^ ^30^ Short in duration with energy concentrated in the lower frequencies Can be present as single, double or triple drums (*T.schlegelli*)^29^ Harmonics absent Formants unknown Dominant frequency (Hz): 30–34^30^ Dominant frequency (Hz)^W^: 51 ± 7^30^ Duration (s): 0.29–0.38^30^		
Growl [Bellow growl, low growl]	A. mississippiensis* ^12^ ^,^ ^16^ ^,^ ^17^ ^,^ ^39^ ^,^ ^40^ *Ca. latirostris* ^21^ *Ca. yacare* ^28^ C. acutus* ^7^, ^16^ *C. mindorensis* ^26^ *C. niloticus* ^16^ *C. porosus* ^14^ *G. gangeticus* ^1^	Demographics	Juvenile & adult, male & female^7^, ^12^, ^16^, ^17^, ^39^, ^40^	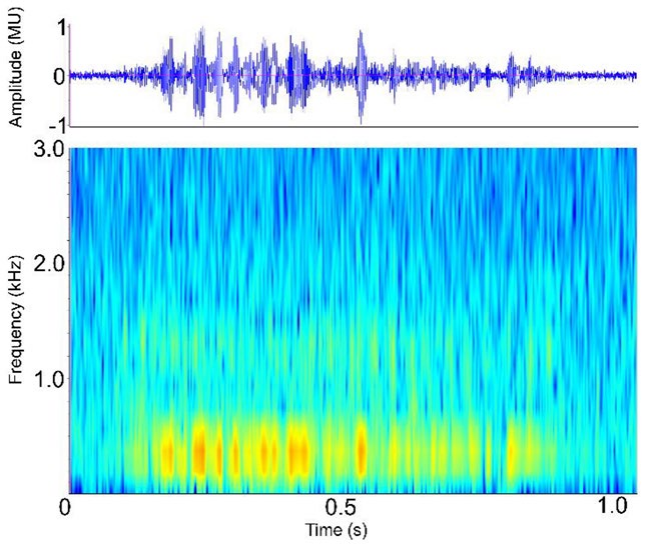	Species: *C. porosus* # Sample rate (Hz): 48,000 Equipment: Microphone Overlap: 50% Brightness: 50% Contrast: 50%
		Acoustic origin	Not specified (likely vocal tract)		
		Production posture/movement	Produced in air, while exhibiting virtually any posture, or locomotion^7^, ^12^, ^16^, ^17^, ^39^, ^40^		
		Description of signal	Deep, guttural, rumbling noise of any volume^17^ ^,^ ^40^		
		Emission context/potential function	Sociality, courtship, aggression, defence, co‐operative feeding^7^, ^12^, ^16^, ^17^, ^39^, ^40^		
		Some defining spectral & temporal characteristics	Frequency modulated with energy concentrated in lower frequencies^7^, ^17^ May display a general concave shape with start end frequencies lower than the centre^7^, ^17^ Harmonics present^7^, ^17^ Formants unknown Dominant frequency (Hz): 100^17^ F0 [START, first note] (Hz): 300^7^ F0 [START, second note] (Hz): 1500^7^ Duration (s): 0.25–0.68^7^, ^17^		
Grunt [Deep grunt]	A. mississippiensis* ^17^ Ca. crocodilus* ^36^ *C. acutus* ^16^ *C. intermedius* ^33^ *C. niloticus* ^16^	Demographics	Adult, female & unknown sex (likely both)^17^ ^,^ ^36^	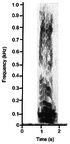	Species: *A. mississippiensis* ^17^, ### Sample rate: Not provided hEquipment: Microphone^17^ FFT: Not provided Window: Not provided Overlap: Not provided Brightness: Not provided Contrast: Not provided Colour scheme: Grayscale
		Acoustic origin	Not specified (likely vocal tract)		
		Production posture/movement	Unknown, but may be locomoting^17^		
		Description of signal	Deep, short, burp‐like^17^ ^,^ ^33^		
		Emission context/potential function	Response to young, courtship^17^ ^,^ ^33^ ^,^ ^36^		
		Some defining spectral & temporal characteristics	Energy concentrated in lower frequencies Harmonics present Formants unknown F0 (Hz): 50^17^ Duration (s): 0.10–1.04^17^ ^,^ ^36^		
Gust	*O. tetraspis* ^30^	Demographics	Adult, sex unknown (paired male & female observed)^30^	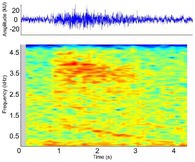	Species: *O. tetraspis* ^*30*^, ## Sample rate (Hz): 1000 Equipment: Hydrophone Overlap: 50% Brightness: 65% Contrast: 50%
		Acoustic origin	Not specified (likely respiratory tract)		
		Production posture/movement	Not specified, measured both in air and underwater30		
		Description of signal	Low frequency wind‐like sound		
		Emission context/potential function	Not specified^30^		
		Some defining spectral & temporal characteristics	Usually occurs with rumbles or drums Decreases in both frequency and energy throughout Harmonics absent Formants unknown Dominant frequency (Hz): 179–259^30^ Dominant frequency (Hz)^W^: 235 ± 44^30^ Duration (s): 1.88–2.34^30^		
Headslap [Head slap, head clap]	A. mississippiensis* ^11^ ^,^ ^16^ ^,^ ^17^ ^,^ ^18^ ^,^ ^39^ ^,^ ^40^ *A. sinensis* ^41^ *Ca. crocodylus* ^14^ *Ca. latirostris* ^14^ *Ca. yacare* ^13^ *C. acutus* ^27^ *C. intermedius* ^27^ ^,^ ^33^ *C. johnstoni* ^14^ *C. moreletii* ^27^ *C. niloticus* ^11^ *C. novaeguineae* ^14^ *C. palustris* ^14^ *C. porosus* ^14^ *C. siamensis* ^14^ *C. suchus* ^14^ *G. gangeticus* ^14^ *M. cataphractus* ^14^ *M. niger* ^14^ *P. palpebrosus* ^14^ *P. trigonatus* ^14^ *T. schlegelii* ^29^	Demographics	Adult male & female (usually male)^11^ ^,^ ^16^ ^,^ ^17^ ^,^ ^18^ ^,^ ^39^ ^,^ ^40^	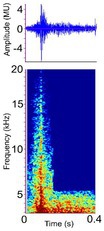	Species: *C. porosus* # Sample rate (Hz): 48,000 Equipment: Microphone FFT: 1024 point Overlap: 95% Brightness: 45% Contrast: 85%
		Acoustic origin	Body—body (upper—lower jaw) & body—object (underside of jaw & head—water surface)^16^ ^,^ ^17^ ^,^ ^18^ ^,^ ^40^		
		Production posture/movement	Produced by raising the head above the surface of the water with the tail arched for balance, the head is lowered forcefully causing the mouth to rapidly close and the lower jaw to make contact with the water surface		
		Description of signal	Loud, percussive, crack‐like sound^11^ ^,^ ^16^ ^,^ ^17^ ^,^ ^18^ ^,^ ^39^ ^,^ ^40^		
		Emission context/potential function	Courtship, aggression, advertisement display, territoriality^11^ ^,^ ^16^ ^,^ ^17^ ^,^ ^18^ ^,^ ^39^ ^,^ ^40^		
		Some defining spectral & temporal characteristics	May be repeated by the same individual^16^ ^,^ ^17^ ^,^ ^18^ ^,^ ^40^ May be produced in a ‘chorus’ of multiple individuals^16^ ^,^ ^17^ ^,^ ^18^ ^,^ ^40^ High energy rapid sound, with a broad bandwidth spike Harmonics absent Formants unknown (and unlikely) Bandwidth (Hz): 50–425^17^ Duration (s): 0.16^17^		
Hiss	A. mississippiensis* ^12^ ^,^ ^16^ ^,^ ^17^ ^,^ ^18^ ^,^ ^40^ A. sinensis* ^41^ *Ca. latirostris* ^21^ Ca. yacare* ^28^ *C. acutus* ^17^ *C. intermedius* ^33^ *C. niloticus* ^17^ *C. porosus* ^42^ *G. gangeticus* ^1^	Demographics	Adult^17^ ^,^ ^41^ (likely all life stages), female^28^ & unknown sex^17^ ^,^ ^41^ (likely both sexes)	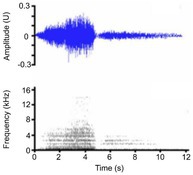	Species: *A. sinensis* ###, ^41^ Sample rate (Hz): 25,000 Equipment: Microphone, tape recorder^41^ Overlap: Not provided Brightness: Not provided Contrast: Not provided Colour scheme: Not provided
		Acoustic origin	Nose^41^ or mouth^16^ (respiratory tract)		
		Production posture/movement	Forceful release of air produced during either or both inhalation or exhalation, with the mouth either open or closed. No upward jet or spray of water is produced, making it distinct from the narial geyser^12^ ^,^ ^16^ ^,^ ^17^ ^,^ ^18^ ^,^ ^28^ ^,^ ^40^ ^,^ ^41^		
		Description of signal	Long, soft, ‘SSSSSS’ sound^16^ ^,^ ^17^ ^,^ ^18^ ^,^ ^41^		
		Emission context/potential function	Aggression, defence, courtship^12^ ^,^ ^16^ ^,^ ^17^ ^,^ ^18^ ^,^ ^28^ ^,^ ^40^ ^,^ ^41^		
		Some defining spectral & temporal characteristics	May be produced as part of a display with bubbles, or narial geysering^16^ ^,^ ^17^ ^,^ ^18^ Properties vary depending on context or threat level^28^ ^,^ ^41^ Harmonics absent^28^ Formants unknown Noisy, broad bandwidth sound lacking melodic structure^28^ Dominant frequency (Hz): 194 ± 40.02^41^ Intercall interval (s): 0.26 ± 0.06^41^ Duration (s): 0.40–10.31^16^ ^,^ ^28^ ^,^ ^41^		
Moan	T. schlegelii* ^29^	Demographics	Adult, unknown sex (paired male & female observed)^29^	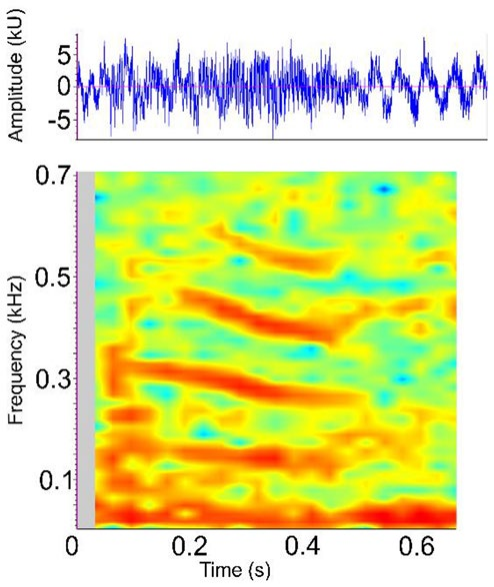	Species: *T. schlegelii* ^29^, ## Sample rate: 4000 Hz Equipment: Hydrophone Overlap: 50% Brightness: 60% Contrast: 50%
		Acoustic origin	Not specified (likely vocal tract)		
		Production posture/movement	Not specified, measured only underwater		
		Description of signal	A short sigh‐like sound that decreases in frequency		
		Emission context/potential function	Courtship^29^		
		Some defining spectral & temporal characteristics	Multiple downsweeps^29^ Produced rarely^29^ Harmonics present^29^ Formants unknown Distinct from the distress call ‘moan’ referred to in the literature Bandwidth (Hz)^W^: 306 ± 92^29^ Dominant frequency (Hz)^W^: 312 ± 119^29^ F0 (Hz)^W^: 141 ± 35^29^ Duration (s)^W^: 0.63 ± 0.14^29^		
Moo	A. sinensis* ^41^	Demographics	Adult & unknown life stages, unknown sex (grouped individuals of both sexes observed)^41^	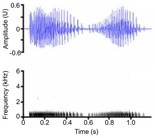	**Species:** *A. sinensis* ###, ^41^ **Sample rate:** 25,000 Hz **Equipment:** Microphone^41^ **Overlap:** Not provided **Brightness:** Not provided **Contrast:** Not provided **Colour scheme:** Not provided
		Acoustic origin	Not specified (likely vocal tract)		
		Production posture/movement	Produced in air with the head at an oblique angle to the surface of the water, with the tail arched^41^		
		Description of signal	Not available		
		Emission context/potential function	Aggression, territoriality/conspecifics display avoidance^41^		
		Some defining spectral & temporal characteristics	Often produced before a headslap^41^ Two sounds present, varying in amplitude^41^ Harmonics & formants unknown Dominant frequency (Hz): 273 ± 57.01^41^ Duration (s): 1.03 ± 0.27^41^		
Moo‐1	*O. tetraspis* ^30^	Demographics	Adult, unknown sex (paired male & female observed)^30^	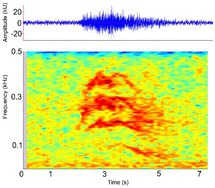	Species: *O. tetraspis* ^*30*^, ## Sample rate (Hz): 1000 Equipment: Hydrophone Overlap: 50% Brightness: 60% Contrast: 50%
		Acoustic origin	Not specified (likely vocal tract)		
		Production posture/movement	Not specified, measured both in air and underwater30		
		Description of signal	A groan‐like sound that increases in volume and frequency toward the centre of the call, and decreases in frequency at the end of the call		
		Emission context/potential function	Not specified		
		Some defining spectral & temporal characteristics	Produced singularly, or in a sequence of up to five^30^ Produced rarely^30^ Frequency modulated upsweep, followed by a frequency modulated downsweep^30^ Harmonics present^30^ Formants unknown Dominant frequency (Hz): 272–313^30^ Dominant frequency (Hz)^W^: 224 ± 50^30^ Duration (s): 2.91–3.14^30^		
Pop	*G. gangeticus* ^1^	Demographics	Adult male^1^	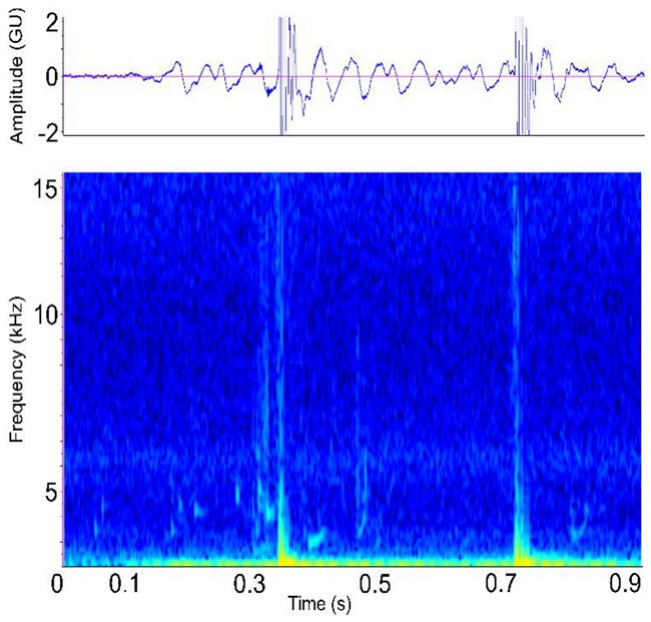	Species: *G. gangeticus* ^1^, ## Sample rate (Hz): 44,100 Equipment: Hydrophone Overlap: 50% Brightness: 50% Contrast: 50%
		Acoustic origin	Produced by the backflow of water into the ghara and nasal tract, in conjunction with a jaw clap^1^. Non‐vocal acoustic signal^1^		
		Production posture/movement	Produced underwater only, in conjunction with a jaw clap (rapid closing of the jaws to produce a percussive sound)^1^		
		Description of signal	Sudden, hollow sounding percussive signal^1^		
		Emission context/potential function	Alert, patrol, individual identity^1^/conspecifics orient toward^1^		
		Some defining spectral & temporal characteristics	Temporal characteristics vary by context & individuality^1^ Produced singularly, or in sequences of up to three^1^ Always preceeded by a sub‐audible vibration and produced as part of a breathing display^1^ Rapid, high energy spike shaped sound. May be performed with bubbling or other signals^1^ Harmonics & formants unknown (and unlikely) Peak frequency (Hz)^W^: 2743.86–2831.63^1^ Duration [first pop] (s)^W^: 0.026–0.029^1^ Duration [second pop] (s)^W^: 0.026–0.028^1^ Inter‐pop‐interal 1 (s)^W^: 0.334–0.336^1^		
Rumble	*O. tetraspis* ^30^ *T. schlegelii* ^29^	Demographics	Adult, sex unknown (paired male & female observed)^29^ ^,^ ^30^	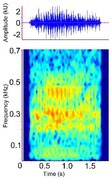	Species: *T. schlegelii* ^29^, ## Sample rate (Hz): 4000 Equipment: Hydrophone Overlap: 50% Brightness: 60% Contrast: 60%
		Acoustic origin	Not specified (likely vocal tract)		
		Production posture/movement	Not specified, measured both in air and underwater^29^ ^,^ ^30^		
		Description of signal	A distinct vibration‐like sound		
		Emission context/potential function	Not specified^30^. Courtship^29^		
		Some defining spectral & temporal characteristics	Strong frequency modulation Harmonics present^29^ ^,^ ^30^ Formants unknown Bandwidth (Hz)^W^: 523 ± 168^29^ Dominant frequency (Hz): 35–51^30^ Dominant frequency (Hz)^W^: 47–164^29^ ^,^ ^30^ Duration (s): 1.78–2.07^29^ ^,^ ^30^ Duration (s)^W^: 1.78 ± 0.48^29^		
Sub‐audible vibration [SAV, infrasound, inaudible bellow]	*A. mississippiensis* ^16^ ^,^ ^17^ ^,^ ^39^ *A. sinensis* ^14^ *Ca. crocodylus* ^14^ *Ca. latirostris* ^14^ *Ca. yacare* ^14^ *C. acutus* ^16^ *C. intermedius* ^33^ *C. johnstoni* ^14^ *C. moreletii* ^14^ *C. niloticus* ^16^ *C. novaeguineae* ^14^ *C. palustris* ^14^ *C. porosus* ^14^ *C. rhombifer* ^14^ *C. siamensis* ^14^ *C. suchus* ^14^ *G. gangeticus* ^1^ *M. cataphractus* ^14^ *M. niger* ^14^ *O. tetraspis* ^14^ *P. palpebrosus* ^14^ *P. trigonatus* ^14^ *T. schlegelii* ^14^	Demographics	Adult male^39^	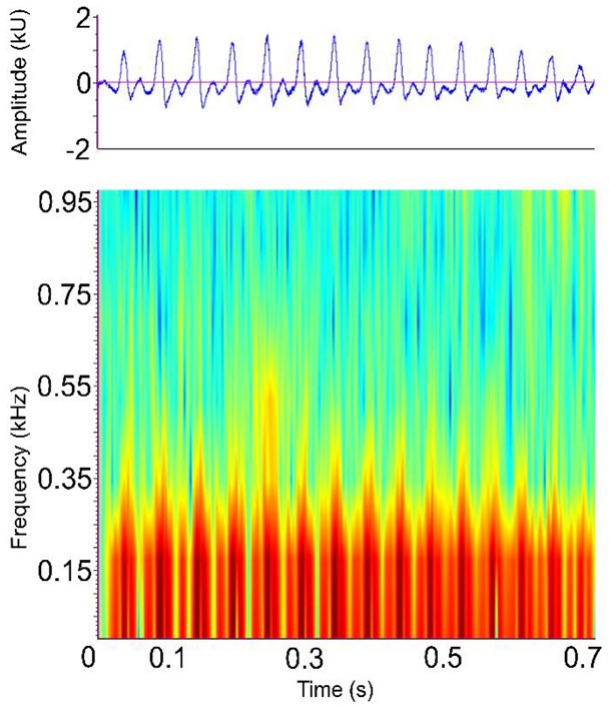	Species: *A. mississippiensis* ## (Jensen et al. 2024) Sample rate (Hz): 44,100 Equipment: Microphone Overlap: 50% Brightness: 50% Contrast: 50%
		Acoustic origin	Resonance of the inflated body cavity^14^		
		Production posture/movement	The animal visibly tenses with its back exposed above the water surface. Very small pulsations are emitted from the torso and may be visible on the skin. Small water droplets bouncing across the animals back, or small vibrating waves emanating from the torso of the animal may also be visible along the water surface (faraday waves). Often produced while the body cavity is inflated and the head is oblique with the tail arched^14^ ^,^ ^16^ ^,^ ^17^ ^,^ ^33^ ^,^ ^39^		
		Description of signal	While not audible to the human ear, the signal is thought to emit infrasound1. Rattle‐like sounds similar to rain drops may be heard from water droplets created by faraday waves		
		Emission context/potential function	Advertisement, aggression, territoriality, courtship^14^ ^,^ ^17^. patrol/alert1		
		Some defining spectral & temporal characteristics	Often produced immediately prior to bellows or headslaps^14^ ^,^ ^16^ ^,^ ^17^ ^,^ ^33^ ^,^ ^39^ Produced prior to the pop^1^ Duration variable based on how frequently the signal is performed (duration decreases with time)^39^ Energy concentrated in very low frequencies (infrasound)^14^ ^,^ ^16^ ^,^ ^17^ ^,^ ^39^ Harmonics & formants unknown Duration [first in a bout] (s): 1.35^39^ Duration [fifth in a bout] (s): 0.55^39^		
Toot	*A. sinensis* ^41^	Demographics	Adult & unknown life stages, unknown sex (grouped individuals of both sexes observed)^41^	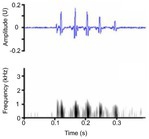	Species: *A. sinensis* ###, ^41^ Sample rate (Hz): 25,000 Equipment: Microphone^41^ Overlap: Not provided Brightness: Not provided Contrast: Not provided Colour scheme: Not provided
		Acoustic origin	Nose^41^		
		Production posture/movement	Produced by the nares, with the mouth under the surface of the water^41^		
		Description of signal	Not available		
		Emission context/potential function	Courtship^41^		
		Some defining spectral & temporal characteristics	Distinct pulses with energy concentrated vertically in the centre of each pulse Amplitude decreases with each successive pulse. Harmonics & formants unknown Dominant frequency (Hz): 186 ± 60.50^41^ Duration (s): 0.217 ± 0.06^41^		
Whine [Whining]	*A. sinensis* ^41^	Demographics	Adult & unknown life stages, unknown sex (grouped individuals of both sexes observed)^41^	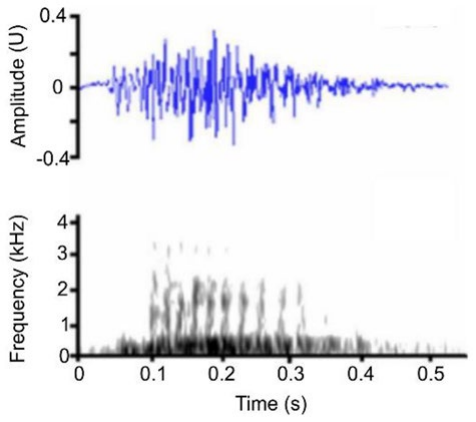	Species: *A. sinensis* ###, ^41^ Sample rate (Hz): 25,000 Equipment: Microphone^41^ Overlap: Not provided Brightness: Not provided Contrast: Not provided Colour scheme: Not provided
		Acoustic origin	Not specified (likely vocal tract)		
		Production posture/movement	Produced by smaller individuals during retreat (locomote away or submerge) from an agonistic encounter with a larger individual^41^		
		Description of signal	Not available		
		Emission context/potential function	Submission/defence^41^		
		Some defining spectral & temporal characteristics	Series of rapidly repeated pulses with energy concentrated in lower frequencies Harmonics & formants unknown Dominant frequency (Hz): 236 ± 53.06^41^ Duration (s): 0.36 ± 0.08^41^		

*Note:* Representative spectrograms have been created using the optimal settings for visualising each acoustic signal; therefore frequency and time scales may vary. Spectrograms were generated using 256‐point fast Fourier transform (FFT), Hann window, and ‘jet’ colour scheme unless otherwise specified. Alternate names from the literature are provided where applicable for each acoustic signal described. Species in which signals have been observed are distinguished from those in which signals were measured based on this review's criteria as indicated with an asterisk beside the species name. Where available, descriptions of associated behavioural postures, sound characteristics, and key acoustic parameters are included. Values for defining spectral and temporal characteristics represent either the mean ± SD where just one mean exists in the dataset, or the range of mean values where multiple means exist in the dataset. Defining spectral and temporal characteristics provided here are not exhaustive and include select parameters only. All parameters measured for each species, sex, context and life stage are available in Supporting Information Table S4. All parameters provided were measured in air, unless accompanied by ^W^(superscript W) which indicates the parameter was measured underwater.

^#^ Spectrogram generated using Raven Pro V1.6 by Flores, S (Unpublished data).

^##^ Spectrogram generated using Raven Pro V1.6 using Supporting Information provided in the cited publication, or directly from the corresponding author.

^###^ Spectrogram adapted from the original article using INKSCAPE V. 3 (2023).

^1^ Ajji and Lang (2025).

^2^ Bollinger (2019).

^3^ Bonke et al. (2015).

^4^ Boucher et al. (2020).

^5^ Brazaitis (1968).

^6^ Brazaitis and Watanabe (2011).

^7^ Campbell (1973).

^8^ Chabert et al. (2015).

^9^ Chabrolles et al. (2017).

^10^ Compton (1981).

^11^ Dinets (2011).

^12^ Dinets (2010).

^13^ Dinets (2013a).

^14^ Dinets (2013b).

^15^ Garrick and Garrick (1978).

^16^ Garrick and Lang (1977).

^17^ Garrick et al. (1978).

^18^ Herzog and Burghardt (1977).

^19^ Hunt and Watanabe (1982).

^20^ Kofron (1991).

^21^ Prystupczuk et al. (2019).

^22^ Reber et al. (2015).

^23^ Reber et al. (2017).

^24^ Riede et al. (2011).

^25^ Roberto and Botero‐Arias (2013).

^26^ Schneider et al. (2014).

^27^ Senter (2008).

^28^ Sicuro et al. (2013).

^29^ Staniewicz et al. (2022).

^30^ Staniewicz et al. (2023).

^31^ Thévenet et al. (2022).

^32^ Thévenet et al. (2023).

^33^ Thorbjarnarson and Hernandez (1993).

^34^ Todd (2007).

^35^ Vergne et al. (2007).

^36^ Vergne et al. (2009).

^37^ Vergne et al. (2011).

^38^ Vergne et al. (2012).

^39^ Vliet (1989).

^40^ Walsh et al. (2022).

^41^ Wang et al. (2007).

^42^ Webb et al. (1977).

**FIGURE 2:**
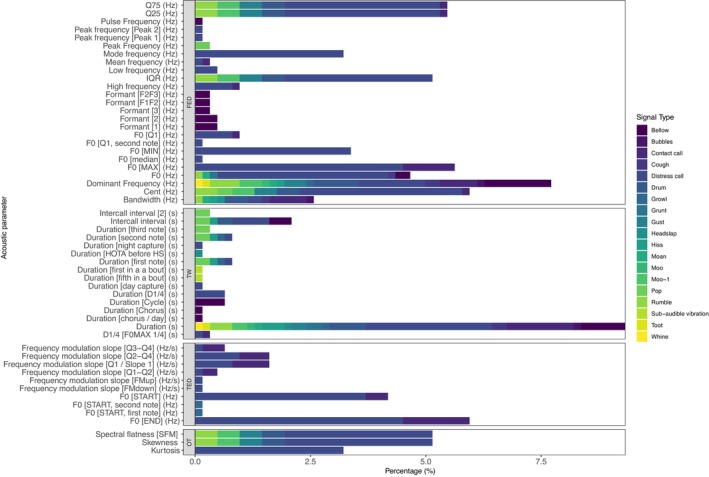
Acoustic parameters included in the dataset presented as a percentage of the total number of parameters observed. Parameters are grouped by category and coloured by the acoustic signal measured in the dataset. Categories: FED, frequency energy distribution; OT, other; TED, time energy distribution; TW, time window.

For the 623 total observations collated (Supporting Information Table S4) we were able to categorise 54 distinct acoustic parameters, most of which (55.0%, *n* = 343) were related to FED. Parameters measuring time window (TW), time energy distribution (TED), and other measurements (OT) comprised just 16.4% (*n* = 102), 15.1% (*n* = 94) and 13.5% (*n* = 84), respectively (Figure 2). Duration (s) was the most commonly measured parameter (9.3%, *n* = 58), followed by dominant frequency (Hz) (7.7%, *n* = 48) and fundamental frequency at the end of the measured signal (F0 [END] (Hz)) (5.9%, *n* = 37). There was a low degree of consistency in the use of acoustic parameters when measuring crocodylian calls, with the majority (67.9%, *n* = 36) representing < 1% each within the dataset.

### 3.3 Parameter Comparison Across Species, Sex and Life Stage

**FIGURE 3:**
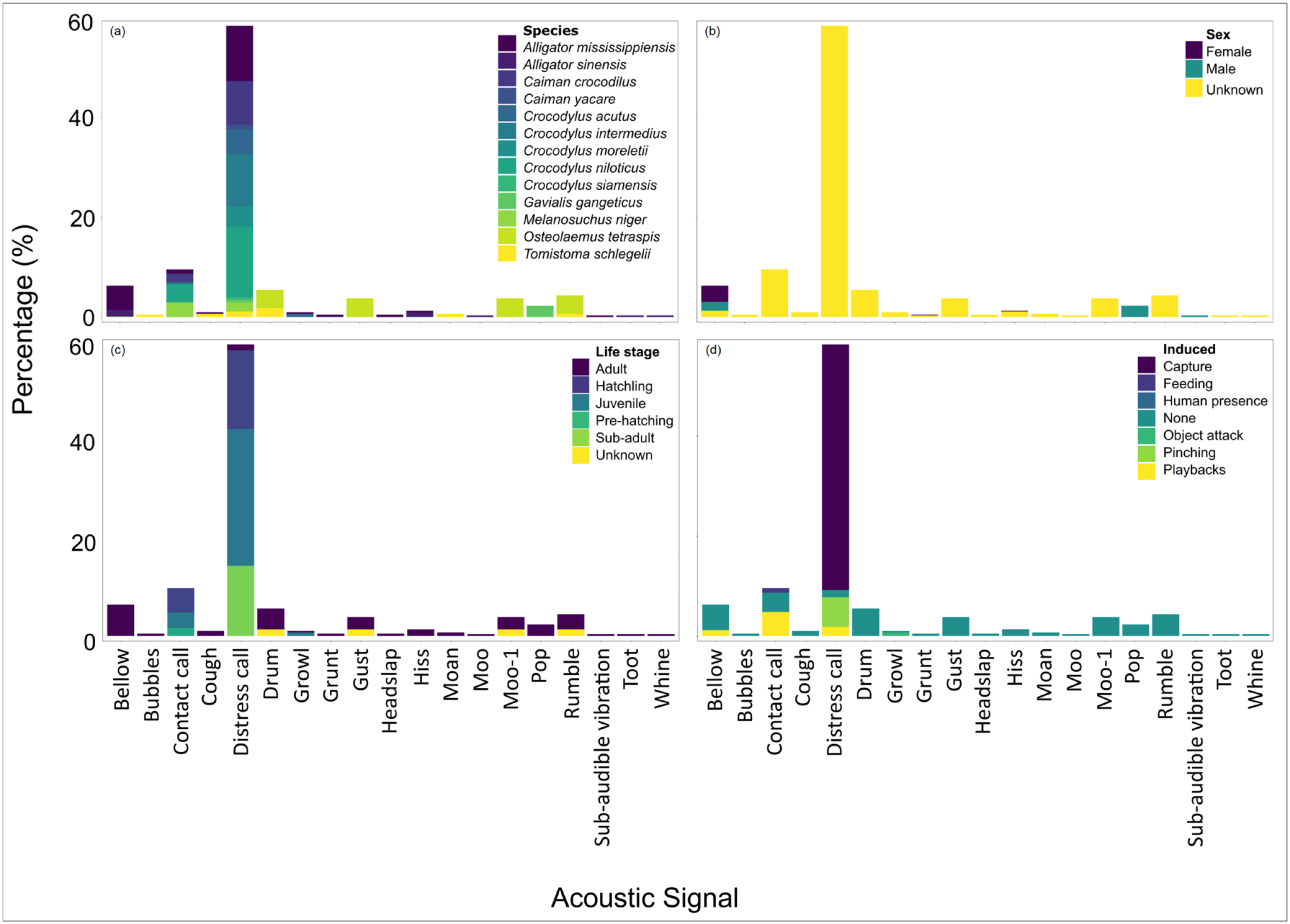
Plots showing percentages of each acoustic signal measured within the dataset, coloured by (a) species, (b) sex, (c) life stage (d) method of call induction (i.e., how the animal was encouraged to produce the acoustic signal measured).

Most parameters (92.2%, *n* = 573) were measured from individuals of unknown sex, with 4.3% (*n* = 27) and 3.5% (*n* = 22) measured from known males and females, respectively (Figure 3b). Bellows were the only call type examined in male and female individuals of known sex (Figure 3b). Life stages were more evenly represented, with juveniles accounting for 31.0% (*n* = 193), adults 27.7% (*n* = 172), hatchlings 20.6% (*n* = 128), sub‐adults 14.0% (*n* = 87) and pre‐hatching 1.6% (*n* = 10) (Figure 3c). Unknown life stages only accounted for 5.1% (*n* = 32) of the dataset. As above, the distress call was the best‐represented call type across life stages, with measurements of pre‐hatching calls the only life stage not measured (Figure 3c).

Most call measurements were recorded in response to a threat (49.7%, *n* = 309), followed by calls made in an unknown context (14.8%, *n* = 92) and then those emitted during courtship (11.1%, *n* = 69). More than half of the dataset (64.2%, *n* = 400) represents measurements made by calls that were induced, with the majority of these made by capture of an individual (49.0%, *n* = 305), all of which were emitted as distress calls (Figure 3d). For this reason, we start with their comparisons, before describing the less commonly measured signals.

#### 3.3.1 Distress Call

**FIGURE 4:**
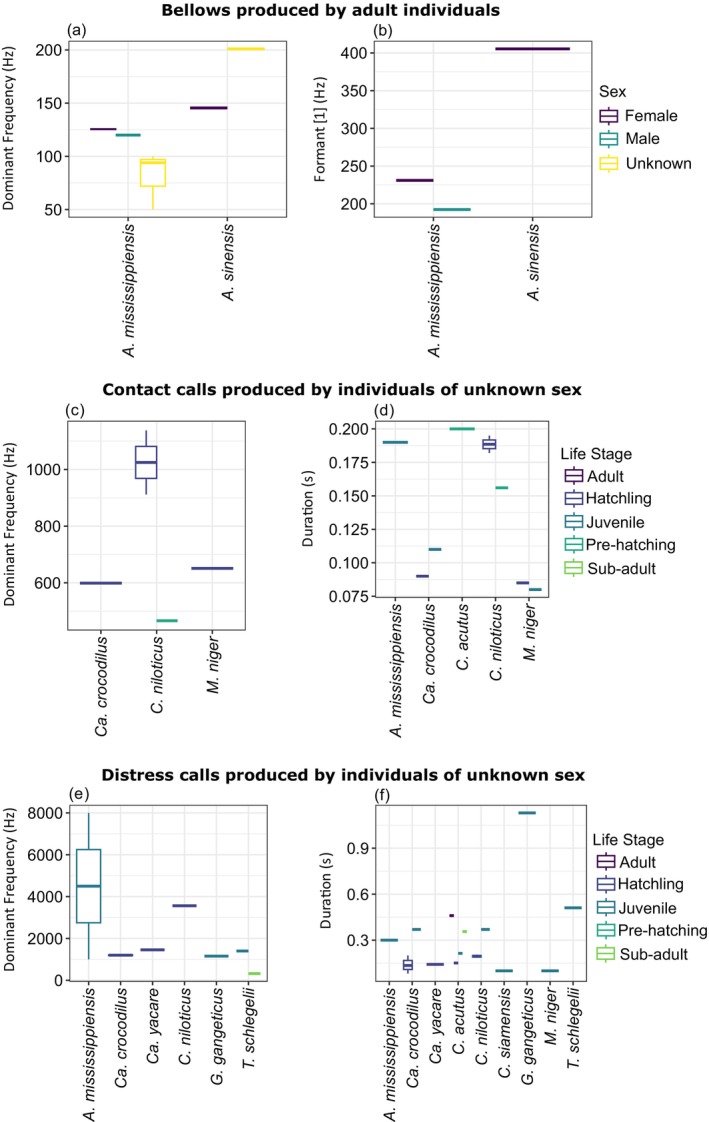
Boxplots of species, sex and life stage variation in the mean values of acoustic signal parameters in the dataset. Plots (a) and (b) display mean values for bellows produced by adults, for (a) dominant frequency (DF) and (b) the first measured formant (Formant [1]). Plots (c) and (d) display the mean values for contact calls produced for (c) the dominant frequency (DF) and (d) call duration. Plots (e) and (f) display the mean values for distress calls produced by individuals of unknown sex, for (e) the dominant frequency (DF) and (f) the call duration. (Additional comparisons can be made by utilising Supporting Information Table S4 which includes the full dataset).

All distress call measurements reported in the literature were measured in air and produced by individuals of unknown sex. We found variation both across and within species for virtually all parameters measured. Notably, distress calls produced by juvenile American alligators had the highest dominant frequency but one of the lowest centre frequency measurements when compared to the five other species measured (Figure 4e). Distress calls produced by American alligators also had a higher fundamental frequency at the start of the call (F0 [start]) but a lower interquartile range (IQR) and Q75 (both measured across the entire call), when compared to the five other species. All species produced distress calls with similar durations (Figure 4f) and fundamental frequency (when measured across the entire call), apart from gharials, whose calls were longer in duration and higher in fundamental frequency than all other species.

#### 3.3.2 Contact Call

Measurements of contact calls in the literature were all done in air and were produced by individuals of unknown sex. When comparing across species we found the Nile crocodile had higher frequencies and longer durations across a range of parameters measured (bandwidth, dominant frequency, duration, fundamental frequency) than the spectacled caiman ( *Caiman crocodilus* , Linnaeus 1758) and the black caiman (*Melanosuchus niger*, Spix 1825) (Figure 4c). Duration was measured in five species for the contact call and showed that the American crocodile ( *Crocodylus acutus* , Cuvier 1807) had the longest duration and the black caiman the shortest (Figure 4d). Fundamental frequency when measured at the end of the contact call (F0 [end]) was also much higher in the American crocodile than those of the four other species measured. Although the contact call was measured across three life stages (hatchling, juvenile and pre‐hatching) observations were mostly for hatchling individuals preventing meaningful comparisons of these parameters across species for juvenile and pre‐hatching individuals.

#### 3.3.3 Bellow

Bellows produced in air by adult Chinese alligators had a higher dominant frequency than those produced by adult American alligators regardless of sex (Figure 4a), and adult male American alligators produced calls longer in duration than conspecific female adults. We also found that both adult male and female American alligators produce calls longer in duration than those of adult Chinese alligators or black caiman (although the sex of the calling individuals was unknown for these latter two species. For American alligators, the duration of bellow cycles was longer in males than in females, and formant frequency was lower in males than in females (Figure 4b).

#### 3.3.4 Drums, Grunts, Hisses and Rumbles

Other call types (drums, grunts, hisses and rumbles) varied in the amount and type of data available, allowing only limited comparisons. Drum calls could only be compared between species underwater, with the dominant frequency of African dwarf crocodile signals found to be much higher than that of false gharials. While grunts were measured in air across two species (American alligator and spectacled caiman), the only parameter noted was duration, with grunts produced by adult American alligators (unknown sex) being longer than those produced by adult (female) spectacled caimans. Adult Chinese alligator (unknown sex) hisses were much longer in duration than those of adult (female) Yacare caiman ( *Caiman yacare* , Daudin 1801), or adult American alligator (unknown sex) adults. The rumble acoustic signal was measured in both air and underwater across two species; however the only parameter available for comparison across the same media was dominant frequency measured underwater, which we found was much higher when produced by false gharials than by African dwarf crocodiles.

**FIGURE 5:**
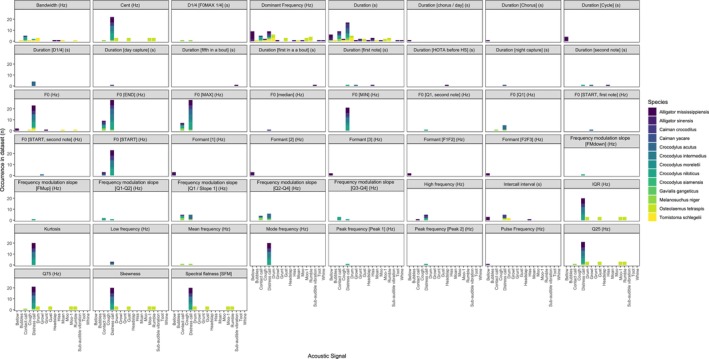
Each plot displays the occurrence of an acoustic parameter measured in this dataset, with bar heights representing occurrence by signal type and colours indicating the species measured. Missing bars indicate that the parameter was not measured for that signal type.

Comparisons of parameters measured for each signal were limited, as few acoustic signals were measured using the same parameters across species, where both sex and life stage of the signalling individual were known (Figure 5).

Since most measurements were taken from recordings made in air, this also precluded our ability to compare many underwater signals, i.e., bellows from American alligators; pops from gharials ( *Gavialis gangeticus* , Gmelin 1789); bubbles, coughs, drums, and moans from false gharials; drums and moo‐1 s from African dwarf crocodiles. Although the cough signal was recorded and measured in two species, these were in different media (i.e., American alligator in air, and false gharial in water), preventing comparison. The growl acoustic signal was measured in air across two species (American alligator and American crocodile). However, these were not comparable since none of the same parameters were measured.

## 4 Discussion

This review summarises the current state of peer‐reviewed, published research on crocodylian acoustic signals. In the 22 papers identified in our review there are detailed descriptions of 19 acoustic signal types from 13 of the 28 extant crocodylian species. Most publications measured distress or contact calls in young and focused on American alligators and/or Nile crocodiles. For many of these studies, the identity and sex of the calling individual were unknown, likely due to the challenges of collecting this information underwater or the need for invasive techniques. The available context on data collection was also limited as many studies did not explicitly investigate this. This lack of consistency made comparisons of the mean values of acoustic signals between species, life stages, sex and context challenging. We provide guidance into further studies seeking to investigate acoustic signalling in crocodylians, and the implications of these findings for passive acoustic monitoring to inform and mitigate human‐crocodile conflict.

### 4.1 Focuses in the Published Literature and Knowledge Gaps

Despite the published studies describing over 600 measurements of crocodylian acoustic signal parameters, there were clear signal types, parameters and even species that were highly represented and others that were not. We found studies predominantly focused on the American alligator and Nile crocodile, with only 13 species having had acoustic signals measured. This leaves over half of the extant crocodylian species entirely unresearched in this field. Considering the threatened status of many of these species, and that conservation and management rely heavily on knowledge about behavioural and population dynamics, there is an unmistakable need for documentation of their acoustic repertoires and associated behaviours.

The uneven documentation of crocodylian acoustic repertoires likely reflects a combination of factors, including access to animals, country economic status, safety and logistical issues associated with working with larger individuals, and disparities in economic resources allocated to conservation and scientific enquiry. For example, Speight et al. (2025) demonstrate an uneven distribution of studies based on IUCN threat status, gross domestic product per capita, and taxonomic group, with notable biases toward ‘Least Concern’ species, studies conducted in the United States and a general neglect of reptile groups. Although a comprehensive analysis of national economic status is beyond the scope of this paper, we acknowledge that research effort to document crocodylian species repertoires may be disproportionately concentrated in regions where species are less threatened, due to more readily available funding—a pattern previously identified in the literature (Moura and Jetz 2021). This bias is particularly relevant for crocodylians, as documenting acoustic signals produced by cryptic apex predators across multiple sound sources and media, including aquatic environments with low visibility and restricted access, presents inherent methodological challenges.

Efforts to measure, analyse and compare crocodylian species have thus far been sporadic, with no full repertoire existing for any species, especially with respect to contexts and underwater signals. Documentation of full repertoires would not only help provide important data for training machine learning models in acoustic monitoring, but also provide insights into their evolutionary history, enabling the construction of an accurate acoustic phylogeny for this clade, even potentially for extinct species. This is particularly relevant for underwater acoustic signals, as only four studies (Todd 2007; Staniewicz et al. 2022, 2023; Ajji and Lang 2025) have explicitly measured these for any crocodylian species worldwide. Acoustic signals are detected by individuals differently in air (by ear) than underwater (likely by bone conduction) (Higgs et al. 2002), which results in a broader range of frequencies being detected in air, but over longer propagation distances underwater (Higgs et al. 2002; Todd 2007). This variation in how signals are propagated across different media suggests that underwater signals, particularly those of low frequencies, may serve distinct ecological functions. For example, in long‐range communication or mediation of territories to prevent costly conflict in aquatic environments (Dinets 2013a; Garrick and Lang 1977). The lack of description of underwater acoustic signals thus represents a significant gap in the literature, and limits our understanding of crocodylian communication systems and their underwater social behaviours.

Most studies included in this review targeted calls made in a threat context (50.8%), whereby calls were induced primarily by capture. Furthermore, these distress calls were produced predominantly by hatchling or juvenile individuals. Studies in other taxa such as birds (Stowell et al. 2017), mammals (Feighny et al. 2006; Caselli et al. 2014), amphibians (Toledo et al. 2015) and arthropods (Fukutomi and Ogawa 2017; Dobai et al. 2018; Kerchev 2019) suggest the context of acoustic signal production is an important factor affecting the parameters of acoustic signals measured. The acoustic signals produced by crocodylians in response to threat are likely to generate a different motivational state when compared to those produced in response to other stimuli such as benign social interactions (Köhler et al. 2017; Dorph and McDonald 2017). Here, we were unable to compare differences in the mean values of measurements of calls based on contexts due to the high degree of variation. In other studies, the acoustic parameters of crocodylian acoustic signals are affected by habitat (Dinets 2011, 2013a, 2013b), anthropogenic disturbance (Boucher et al. 2020) and signalling location (air vs. water) (Jensen et al. 2024) indicating the importance of context. Context may also include individual state; for example juvenile Nile crocodiles that are sated show less attention to socially relevant acoustic signals in the presence of food odour than those that are non‐sated (Chabrolles et al. 2017). Unfortunately, the above study did not include measurements of the signals produced so it was excluded from our parameter analysis. Examining differences in acoustic signals produced by crocodylians under different contexts, motivational states and methods of induction (e.g., playbacks vs. passive vs. capture), presents significant opportunities to enhance our understanding of crocodylian behaviour overall.

We were unable to compare many signals across species (e.g., bubbles, cough, growl, gusts etc.), despite our efforts to consolidate acoustic signal types and parameter mean values for this purpose. Even for more frequently reported signals such as the contact call and distress call, comparisons were limited due to differences in calling contexts, unknown sex of individuals, and methods used to encourage individuals to produce these calls. Likewise, signals measured in air were (and are) not comparable to the same signals measured underwater, due to both differences in sound propagation and differences in crocodylian acoustic signal perception (Higgs et al. 2002; Papet et al. 2019). Future studies should aim to report on these metrics (sex, context, environment, etc.) when researching acoustic signalling in crocodylians to promote comparisons across studies.

### 4.2 Acoustic Parameter Measurements and Associated Behaviours

Across 13 crocodylian species, over 600 measurements of acoustic parameters were able to be analysed from the literature. Our analysis, while limited in its ability to directly compare many call types according to their parameters because of the variability in the data, was able to consolidate the existing information in such a way that allowed the creation of a standardised and detailed ethogram that we hope will be used in future studies.

Processing acoustic data is a large undertaking requiring a significant time investment. This is especially true when using passive recordings, analysing through different media (e.g., air and water), and where species' repertoires have not been previously well defined. Further challenges arise due to the variety of acoustic software available for processing, the abundance of available measurements to choose from within these programs, and the diversity of ways to visualise these signals. To help streamline future crocodylian acoustic research and assist in identifying, visualising, and classifying acoustic signals, we have included representative spectrograms in our ethogram (Table 3). We specify settings used to generate these spectrograms, since optimal signal visualisation and analysis can differ based on factors such as recording equipment, sample rate etc. To promote consistency in future research, we have also compiled a glossary of terms (Supporting Information Table S6) as a guide. To allow comparisons across species with existing published datasets, and for construction of acoustic phylogenies, we also recommend sampling a specific set of acoustic parameters with known importance to crocodylian behaviour, as follows:

#### 4.2.1 Formant Frequency

Formants are produced from the resonance of the vocal tract and therefore scale with body size (Reber et al. 2017). They have been shown to contain individual identity, and to predict sex in American alligator bellows and likely contain information pertaining to body size and fitness in this species (Jensen et al. 2024).

#### 4.2.2 Call Duration, Intercall Interval, Rate and Syntax

Call duration, intercall interval and call rate show individuality and variation by context in gharial pops, in addition to life stage and sex identification (Ajji and Lang 2025). Differences in acoustic signalling syntax (i.e., the sequence of signal elements) may allow species identification and potentially contain contextual, individual and/or behavioural information (Garrick et al. 1978; Kershenbaum et al. 2016).

#### 4.2.3 Fundamental Frequency (F0) and Contours/Slope

Fundamental frequency, and its shape throughout the call (i.e., contour or slope), including measurements of energy distribution along the spectrum (such as F0 [max]) have consistently been found to contain important information in crocodylian signalling (Vergne et al. 2012; Thévenet et al. 2023; Jensen et al. 2024). For example, the F0 contour of the American alligator bellow is an important identifier of individuality (Jensen et al. 2024). Likewise, the shape of the F0 can help to distinguish meaningful boundaries between graded calls, such as that of the contact and distress call (Reber 2020). Frequency modulation measured as slope along various segments of the contact call can also indicate species (Vergne et al. 2012) or caller size (Chabert et al. 2015), eliciting different responses from receivers. Additionally, the overall shape, or spectral envelope may be an informative parameter in caimans (Thévenet et al. 2023).

#### 4.2.4 Cent/Centroid

The spectral centroid is an important predictor of individuality in the American alligator bellow (Jensen et al. 2024), and size in the contact call of juveniles from various species (Chabert et al. 2015).

#### 4.2.5 Amplitude Modulation

Amplitude modulation is likely achieved through manipulation of tissues above the glottis in American alligators, and conveys individual and body size information in bellows (Jensen et al. 2024). Conversely, amplitude modulation appears to be unimportant in species specificity of juvenile contact calls, suggesting this may not be a universally informative parameter (Vergne et al. 2012).

#### 4.2.6 Call Onset

There is evidence that measurements taken from the first third, and first half of American and Chinese alligator bellows are important in individual identification and producing a behavioural response from conspecifics (Wang et al. 2007; Jensen et al. 2024). This is not necessarily the case for measurements taken at the end of a call.

### 4.3 Influence of Species, Sex, Size and Life Stage

Where comparisons of acoustic signals were possible, we found differences in measured parameters of signals could often be explained by sex, life stage or size of the calling individual. We found species‐specific differences in virtually every call type where comparisons of parameter mean values were possible (bellow, contact call, distress call, drum, grunt, hiss, rumble). However, whether these species‐specific differences are simply a consequence of individual sender morphology, or have been shaped through selection (e.g., to avoid hybridisation) is currently unclear (noting these are not mutually exclusive explanations). Research has shown that some individuals respond similarly to acoustic signals produced by juvenile individuals of different species (Vergne et al. 2012), however this has not been investigated broadly across signal types, species or life stages. Distress calls in particular may be more conserved than other call types (Aubin 1991), for example, and thus variability in these calls may signify urgency more so than individual or species information. Employing playback experiments where sex, body size, species and context are known for both the signalling individual and the conspecific respondent would likely help shed light on how individuals might distinguish call types, individual information, or contexts.

Sex appears to significantly predict some of the acoustic parameters of different call types. For example, calls of male American alligators are longer than those of females. Dominant frequency (along with all formant‐related parameters) in American alligator bellows is also higher in females than that of males, with this trend consistent across studies (Reber et al. 2017; Jensen et al. 2024). Crocodylians exhibit male‐biased sexual dimorphism; thus this may be an example of size‐signal allometry, whereby larger individuals produce lower frequency vocalisations, although this likely varies across species (Martin et al. 2017; López‐Cuamatzi et al. 2020). This size‐frequency relationship is based on whether vocal fold size (the main determinant of frequency) correlates with overall body size, which may not be the case for all species (Reber et al. 2017; Jensen et al. 2024). Whether signals encode sex through other parameters is yet to be investigated. In wild studies, this research is complicated by the fact that it is not often possible to accurately differentiate between sexes, especially in hatchlings, juveniles, or sub‐adults without invasive techniques (e.g., cloacal examination or blood sampling; Grigg and Kirshner 2015). However, determining whether crocodylian acoustic signals encode for sex would enhance our ability to classify individuals using non‐invasive methods, increase our understanding of how acoustic signalling differs by sex, and contribute to our knowledge of sexual selection in crocodylians.

Formant frequency of an acoustic signal, in contrast, is produced from resonant frequencies of the vocal tract, which is constrained and scales according to body size. Thus, this parameter is more generally considered an honest signal of individual size, with larger individuals producing lower frequency formants than their smaller counterparts (Fitch and Hauser 2002; Hodges‐Simeon et al. 2014). Indeed, bellow formant frequencies of Chinese and American alligators are a reliable indicator of body size (Reber et al. 2015, 2017). It is unknown whether formants exist in calls produced by other crocodylian genera however, highlighting a significant gap in our understanding of crocodylian acoustic communication and its evolution and at the same time offering an opportunity for future research.

Frequency‐based parameters associated with body size may also indicate age or life stage, especially if these measurements scale with growth across an individual's lifetime. Behavioural research may set a foundation for this. For example, Nile crocodile mothers have been shown to respond more strongly to the vocalisations of smaller offspring (Chabert et al. 2015). Unfortunately, it is unclear whether this response was dependent on formant frequencies, since these were not measured. Although crocodylians have been shown to exhibit rapid changes in acoustic signals post‐hatch (Russell and Bauer 2021), no studies have specifically measured and compared these features over longer timeframes; thus very little is known about how signals develop or vary across an individual's lifespan. In other taxa, including birds and fish, acoustic signals are known to develop and change with age (Catchpole and Slater 2008; Ladich 2015). In the data collated here, measurements between call types, species and life stages differed to an extent that a general rule of body‐size scaling with age could not be determined for any parameter. Understanding the ontogeny of crocodylian signals and their potential relationships to age, especially in species‐specific studies, would enable further useful information in acoustic monitoring and scientific inquiry.

Future studies on crocodylian acoustic signals should ensure a broad range of acoustic parameters are measured and reported for each signal, including both frequency and time‐based measurements, to enable between and within‐species comparisons. Formant frequency measurements in particular would shed light on whether size‐signal allometry exists in this group (refer to Section 2). Researchers holding existing datasets of crocodylian acoustic signals may also consider reprocessing or sharing their raw datasets to allow this measurement to be extracted, referring to methods described in Reber et al. (2017). Doing so could provide valuable insights without the costs or safety concerns associated with additional data collection.

### 4.4 Additional Considerations

Acoustic signals, especially in crocodylians, are not limited to vocalisations. Non‐vocal acoustic signals such as pops, headslaps, hissing or narial geysering (where individuals send a spout of water from their nares) may also encode contextual or individual information, and warrant further investigation. Hissing in particular is intriguing, as it is unknown whether animals produce this signal via the mouth (Garrick and Lang 1977) or the nose (Wang et al. 2007). Additionally, the contexts for hissing in crocodylians are highly variable and include aggression, defence and courtship (Garrick and Lang 1977; Garrick et al. 1978; Wang et al. 2007; Dinets 2010). Investigating whether signal production varies across different contexts would be valuable.

Recent studies on underwater ‘pop’ signals in gharials provide a notable example of how acoustic research has significantly advanced our understanding of non‐vocal acoustic communication in crocodylians. These are produced as a function of the ghara—the soft tissue narial extrusion found only on adult male gharials (Ajji and Lang 2025). These pops appear to convey identity, behavioural context (e.g., patrolling) and location information, and elicit appropriate and relevant responses by conspecifics. Temporal parameters including duration, intercall interval, and rate were key components identified as being informative in determining identity and context in gharial pops (Ajji and Lang 2025). This is particularly noteworthy as our review highlights a strong research focus on frequency‐based parameters (comprising over half of the dataset), while time‐window and time‐energy parameters were reported far less frequently in the published literature. Future research should ensure temporal parameters of signals are included in analyses, especially when collected in natural behavioural settings. Again, those holding existing datasets meeting these criteria may consider additional analysis to include these measurements, particularly where these datasets have been collected within known contexts and from individuals of known sex and size.

The potential application for monitoring crocodylians through their acoustic signals is significant, and the implementation of this technology could provide a cost‐effective, non‐invasive way of gathering information about individuals and populations. Manually processing the vast amount of data gathered through passive acoustic monitoring can require significant time investment, often not feasible for most researchers (Stowell 2022). However, machine learning techniques to process acoustic data are revolutionising such studies, through the development of techniques including automatic detection, measurement, and the application of species‐specific call recognisers (Bianco et al. 2019; Teixeira et al. 2019; Eichinski et al. 2022; Stowell 2022). Machine learning for call detection and identification has traditionally required a significant amount of pre‐processed data, but recent breakthroughs have demonstrated that less data can be sufficient, depending on research goals (Knight et al. 2020; Eichinski et al. 2022). At this time, recognisers are out of reach for most (if not all) crocodylian species due to significant data gaps that exist at a foundational level (e.g., species' full repertoires, underwater signal data). Given the difficulties of collecting and processing passive acoustic data from these cryptic apex predators, the importance of consistent and transparent access to raw acoustic datasets (and accompanying metadata) cannot be underestimated. This is particularly important for supporting global research efforts, especially in regions with abundant crocodylian populations but limited resources, where many species are threatened.

While a comprehensive methodological framework for quantifying species repertoires or collecting acoustic signalling data is beyond the scope of this review, we recommend that researchers consider the following key factors prior to their investigations. First and foremost, researchers should begin by clearly defining their specific research question as this will guide the design and methodological approach and its implications for signal type and context. Second, researchers should ensure their equipment requirements are tailored to the frequency ranges and sampling media (e.g., equipment to capture low‐frequency signals underwater may differ from that required to capture signals produced in air and within the human hearing range). Third, there should be consideration of the potential impacts of background environmental noise, and internal noise levels of the recorder itself (Kitzes et al. 2025). As crocodylian acoustic signals vary widely in amplitude, from loud signals such as roars and headslaps, to subtle sounds like hisses, bubbles and gusts, certain signals may be masked by background noise. Last is the choice between captive and wild study locations, as each offers distinct advantages and limitations, with behavioural differences across settings potentially influencing signal production (Walsh et al. 2022).

## 5 Conclusions

Published, peer‐reviewed studies to date focus primarily on only a few species, with most extant crocodylians lacking any published acoustic signal data. Additionally, most studies lack measurements of underwater acoustic signals. Full, passively/naturally recorded, baseline species repertoires, including identity information of signallers and receivers, are necessary to advance our understanding of the ecology and evolution of crocodylian acoustic signals as well as overall behaviour. Although data variability precluded our ability to directly compare most signals, we were able to construct a standardised ethogram of each acoustic signal type, including representative spectrograms. We also provide a standardised summary table of measurements, a comprehensive glossary of terms to allow for robust comparisons, and a brief methodological framework to guide future research. We specifically recommend sampling specific acoustic parameters with known importance to crocodylian behaviour including (i) formant frequency, (ii) call duration, intercall interval, rate and syntax, (iii) fundamental frequency and contours/slope, (iv) cent/centroid, (v) amplitude modulation, and (vi) call onset.

Sex, life stage or size of the calling individual may influence parameters differently across acoustic signal types. Males tend to produce lower frequency calls than females, and adults produce longer calls than juveniles. Determining whether crocodylian acoustic signals encode for sex or age would greatly enhance our own ability to classify individuals using non‐invasive methods. While formants are likely to encode for some of these characteristics, playback experiments are necessary to determine how individuals perceive and respond to signal parameters based on environment, context or signaller identity. Further study on other key components of crocodylian acoustic signalling is proposed. Specifically, non‐vocal acoustic signals likely convey important information, and temporal‐based parameters of these, including intercall interval, rate and syntax should be considered in future investigations. Collaborations between researchers, and reanalysis of existing datasets to incorporate standard measurements present scientific opportunities without the risks of further fieldwork. This also has the potential to inform on threatened species in regions with limited resources. With emerging technologies such as machine learning and the development of species‐specific call recognizers, the potential application for passive acoustic monitoring of crocodylians by their acoustic signals is significant. These rely on comprehensive species repertoires and sufficient acoustic data sets, which should be prioritised.

## Author Contributions

**Sonnie A. Flores:** conceptualization (lead), data curation (lead), formal analysis (lead), funding acquisition (lead), investigation (lead), methodology (lead), visualization (lead), writing – original draft (lead), writing – review and editing (equal). **Ross G. Dwyer:** conceptualization (equal), formal analysis (supporting), funding acquisition (supporting), project administration (lead), supervision (equal), visualization (supporting), writing – original draft (supporting), writing – review and editing (supporting). **Stuart Parsons:** conceptualization (supporting), supervision (supporting), writing – original draft (supporting), writing – review and editing (supporting). **Dominique A. Potvin:** conceptualization (equal), data curation (supporting), formal analysis (supporting), funding acquisition (supporting), investigation (supporting), project administration (supporting), supervision (equal), visualization (supporting), writing – original draft (supporting), writing – review and editing (equal).

## Conflicts of Interest

The authors declare no conflicts of interest.

## Supporting information


**Material S1.** LitRv_Data_R.csv: File used for R code to produce statistics and figures.
**Material S2.** CrocsLitRV_R_Code_V4_pub_SFlores.R: R code for production of statistics and figures.
**Material S3.** Photo of an adult female estuarine crocodile ( *Crocodylus porosus* ) on the Daintree River (Queensland, Australia) producing a vocalisation. Photo credit: Mr. David White.

**Table S4.** Summary table of acoustic measurements _all species_Flores et al. 2025.

**Table S5:** Calculations_Flores et al. 2025.

**Table S6:** Glossary of terms_Flores et al. 2025.

**Material S7:** Reference list for supporting information files.

## Data Availability

The data that support the findings of this study is available in the Supporting Information and Appendix.

## Appendix A

Balaguera‐Reina, S.A. et al. (2018) “*Crocodylus intermedius* (errata version published in 2020).” The IUCN Red List of Threatened Species. Accessed October 1, 2025. https://doi.org/10.2305/IUCN.UK.2018‐1.RLTS.T5661A181089024.en.

Balaguera‐Reina, S.A. and Velasco, A. (2019) “*Caiman crocodilus*.” The IUCN Red List of Threatened Species. Accessed October 1, 2025. https://doi.org/10.2305/IUCN.UK.2019‐1.RLTS.T46584A3009688.en.

Bezuijen, M. et al. (2012) “*Crocodylus siamensis*.” The IUCN Red List of Threatened Species. Accessed October 1, 2025. https://doi.org/10.2305/IUCN.UK.2012.RLTS.T5671A3048087.en.

Campos, Z. et al. (2020) “*Caiman yacare*.” The IUCN Red List of Threatened Species. Accessed October 1, 2025. https://doi.org/10.2305/IUCN.UK.2020‐3.RLTS.T46586A3009881.en.

Campos, Z., Magnusson, W.E. and Muniz, F. (2019) “*Paleosuchus trigonatus*.” The IUCN Red List of Threatened Species. Accessed October 1, 2025. https://doi.org/10.2305/IUCN.UK.2019‐1.RLTS.T46588A3010035.en.

Carr, A.N. et al. (2021) “Use of continuous cranial shape variation in the identification of divergent crocodile species of the genus Mecistops,” *Journal of Morphology*, 282(8), pp. 1219–1232. https://doi.org/10.1002/jmor.21365.

Choudhury, B.C. and de Silva, A. (2013) “*Crocodylus palustris*.” The IUCN Red List of Threatened Species. Accessed October 1, 2025. https://doi.org/10.2305/IUCN.UK.2013‐2.RLTS.T5667A3046723.en.

Crocodile Specialist Group (1996) “*Osteolaemus tetraspis*.” The IUCN Red List of Threatened Species. Accessed October 1, 2025. https://doi.org/10.2305/IUCN.UK.2000.RLTS.T13053A3407604.en.

Elsey, R., Woodward, A. and Balaguera‐Reina, S.A. (2019) “*Alligator mississippiensis*.” The IUCN Red List of Threatened Species. Accessed October 1, 2025. https://doi.org/10.2305/IUCN.UK.2019‐2.RLTS.T46583A3009637.en.

Hekkala, E. et al. (2011) “An ancient icon reveals new mysteries: mummy DNA resurrects a cryptic species within the Nile crocodile,” *Molecular Ecology*, 20(20), pp. 4199–4215. https://doi.org/10.1111/j.1365‐294X.2011.05245.x.

Isberg, S. et al. (2019) “*Crocodylus niloticus*.” The IUCN Red List of Threatened Species. Accessed October 1, 2025. https://doi.org/10.2305/IUCN.UK.2019‐1.RLTS.T45433088A3010181.en.

Isberg, S., Balaguera‐Reina, S.A. and Ross, J.P. (2017) “*Crocodylus johnstoni*.” The IUCN Red List of Threatened Species. Accessed October 1, 2025. https://doi.org/10.2305/IUCN.UK.2017‐3.RLTS.T46589A3010118.en.

Jiang, H.X. and Wu, X. (2018) “*Alligator sinensis*.” The IUCN Red List of Threatened Species. Accessed October 1, 2025. https://doi.org/10.2305/IUCN.UK.2018‐1.RLTS.T867A3146005.en.

Lang, J.W., Chowfin, S. and Ross, J.P. (2019) “*Gavialis gangeticus* (errata version).” The IUCN Red List of Threatened Species. Accessed October 1, 2025. https://doi.org/10.2305/IUCN.UK.2019‐1.RLTS.T8966A149227430.en.

Lourenço‐de‐Moraes, R. et al. (2023) “Global conservation prioritization areas in three dimensions of crocodilian diversity,” *Scientific Reports*, 13, p. 2568. https://doi.org/10.1038/s41598‐023‐28413‐6.

Magnusson, W.E., Campos, Z. and Muniz, F. (2019) “*Paleosuchus palpebrosus*.” Accessed October 1, 2025. https://doi.org/10.2305/IUCN.UK.2019‐1.RLTS.T46587A3009946.en.

McMahan, W. et al. (2022) “*Crocodylus rhombifer*.” The IUCN Red List of Threatened Species. Accessed October 1, 2025. https://doi.org/10.2305/IUCN.UK.2022‐2.RLTS.T5670A130856048.en.

Murray, C.M. et al. (2024) “Divergent Morphology among Populations of the New Guinea Crocodile, *Crocodylus novaeguineae* (Schmidt, 1928): Diagnosis of an Independent Lineage and Description of a New Species,” *Copeia*, 107(3), pp. 517–523. https://doi.org/10.1643/CG‐19‐240.

Platt, S.G. et al. (2023) “*Crocodylus moreletii*.” The IUCN Red List of Threatened Species. Accessed October 1, 2025. https://doi.org/10.2305/IUCN.UK.2023‐1.RLTS.T5663A193672551.en.

R Core Team (2024) ‘R: A Language and Environment for Statistical Computing’. Vienna, Austria: R Foundation for Statistical Computing. https://www.R‐project.org/.

Rainwater, T.R. et al. (2022) “*Crocodylus acutus* (amended version of 2021 assessment).” The IUCN Red List of Threatened Species. Accessed October 1, 2025. https://doi.org/10.2305/IUCN.UK.2022‐1.RLTS.T5659A212805700.en.

Ross, J.P. (2000) “*Melanosuchus niger*.” The IUCN Red List of Threatened Species. Accessed October 1, 2025. https://doi.org/10.2305/IUCN.UK.2000.RLTS.T13053A3407604.en.

Shaney, K. et al. (2023) “*Tomistoma schlegelii*.” The IUCN Red List of Threatened Species. Accessed October 1, 2025. https://doi.org/10.2305/IUCN.UK.2023‐1.RLTS.T21981A214287051.en.

Shirley, M.H. (2014) “*Mecistops cataphractus*.” The IUCN Red List of Threatened Species. Accessed October 1, 2025. https://doi.org/10.2305/IUCN.UK.2014‐1.RLTS.T5660A3044332.en.

Siroski, P. et al. (2020) “*Caiman latirostris*.” The IUCN Red List of Threatened Species. Accessed October 1, 2025. https://doi.org/10.2305/IUCN.UK.2020‐3.RLTS.T46585A3009813.en.

Smolensky, N.L., Hurtado, L.A. and Fitzgerald, L.A. (2015) “DNA barcoding of Cameroon samples enhances our knowledge on the distributional limits of putative species of Osteolaemus (*African dwarf crocodiles*),” *Conservation Genetics*, 16(1), pp. 235–240. https://doi.org/10.1007/s10592‐014‐0639‐3.

Solmu, G. and Manolis, C. (2019) “*Crocodylus novaeguineae*.” The IUCN Red List of Threatened Species. Accessed October 1, 2025. https://doi.org/10.2305/IUCN.UK.2019‐2.RLTS.T46591A3010398.en.

Webb, G. et al. (2021) “*Crocodylus porosus*.” The IUCN Red List of Threatened Species. Accessed October 1, 2025. https://doi.org/10.2305/IUCN.UK.2021‐2.RLTS.T5668A3047556.en.

van Weerd, M. et al. (2016) “*Crocodylus mindorensis*.” The IUCN Red List of Threatened Species. Accessed October 1, 2025. https://doi.org/10.2305/IUCN.UK.2016‐3.RLTS.T5672A3048281.en.
