# Supplementary figures and images for: Roars, Rumbles, and Resonance: A Systematic Review and Meta‐Analysis of Crocodylian Acoustic Signals
Source: Ecol Evol. 2026 Jan 22;16(1):e72494. doi: 10.1002/ece3.72494 (PMC12828176; doi:10.1002/ece3.72494)

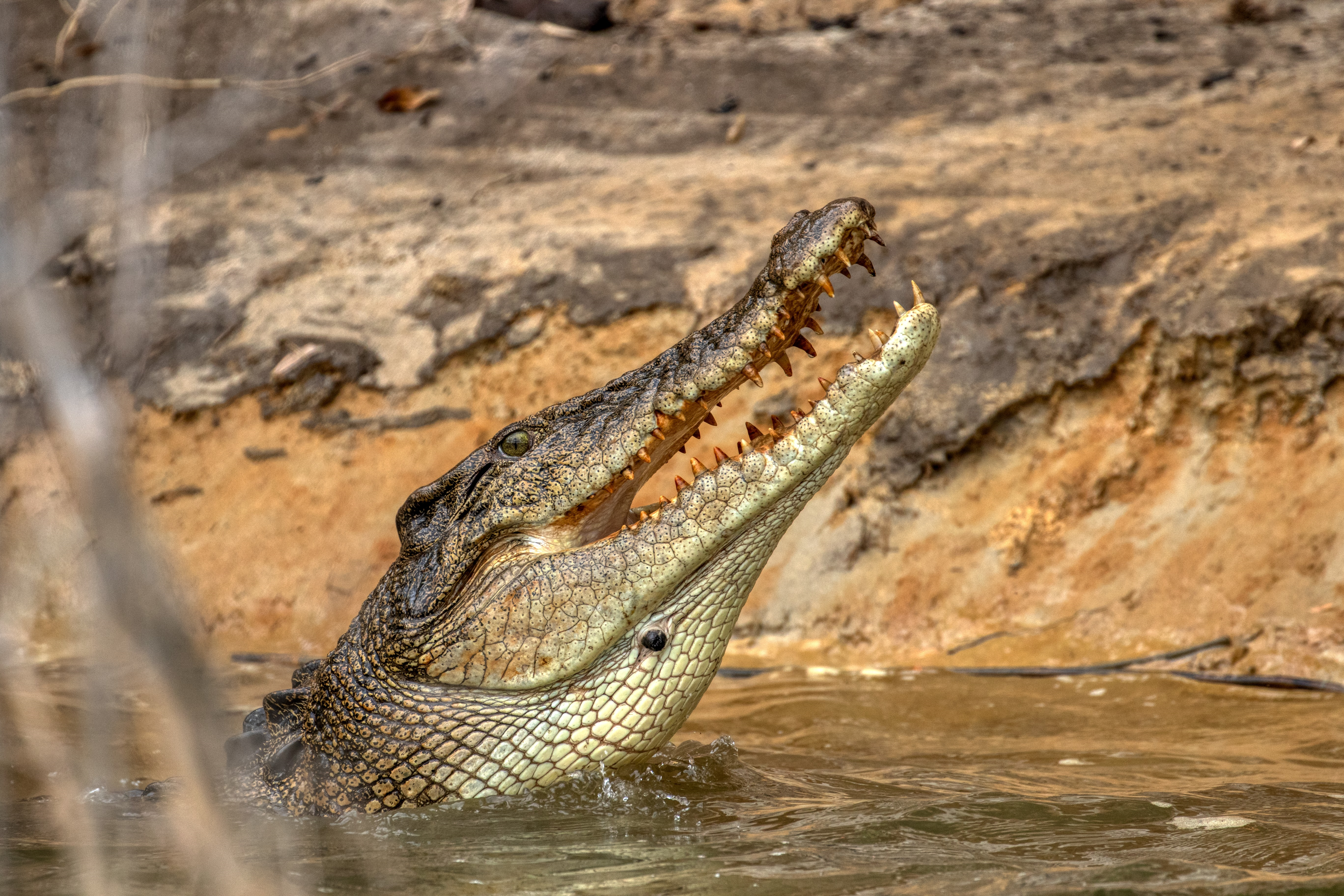

Supplement: Supplementary file 1 — Material S1. LitRv_Data_R.csv: File used for R code to produce statistics and figures. Material S2. CrocsLitRV_R_Code_V4_pub_SFlores.R: R code for production of statistics and figures. Material S3. Photo of an adult female estuarine crocodile ( Crocodylus porosus ) on the Daintree River (Queensland, Australia) producing a vocalisation. Photo credit: Mr. David White. [file ECE3-16-e72494-s005.zip › S3. Credit David White.png]
