# Supplementary material for: Roars, Rumbles, and Resonance: A Systematic Review and Meta‐Analysis of Crocodylian Acoustic Signals
Source: Ecol Evol. 2026 Jan 22;16(1):e72494. doi: 10.1002/ece3.72494 (PMC12828176; doi:10.1002/ece3.72494)
Supplement: Supplementary file 5 — Material S7: Reference list for supporting information files. [file ECE3-16-e72494-s003.docx]

**Supporting Information Reference Lists for Flores et al., (2025)**

Roars, Rumbles, and Resonance: A Systematic Review and Meta- Analysis of Crocodylian Acoustic Signals

**Table S4. Summary table of acoustic measurements**

Ajji M, J., & Lang, J. W. (2025). Gharial acoustic signaling: Novel underwater pops are temporally based, context-dependent, seasonally stable, male-specific, and individually distinctive. *Journal of Anatomy*. https://doi.org/10.1111/joa.14171

Bonke, R., Whitaker, N., Roedder, D., & Boehme, W. (2015). Vocalizations in two rare crocodilian species: A comparative analysis of distress calls of Tomistoma schlegelii (Müller, 1838) and Gavialis gangeticus (Gmelin, 1789). *North-Western Journal of Zoology*, *11*(1), 151–162.

Boucher, M., Tellez, M., & Anderson, J. T. (2020). Differences in distress: Variance and production of American Crocodile (*Crocodylus acutus*) distress calls in Belize. *Ecology and Evolution*, *10*(18), 9624–9634. https://doi.org/10.1002/ece3.6556

Campbell, H. W. (1973). Observations on acoustic behaviour of crocodilians. *Zoologica*, *58*(1), 11.

Chabert, T., Colin, A., Aubin, T., Shacks, V., Bourquin, S. L., Elsey, R. M., Acosta, J. G., & Mathevon, N. (2015). Size does matter: Crocodile mothers react more to the voice of smaller offspring. *Scientific Reports*, *5*(1), 15547–15547. https://doi.org/10.1038/srep15547

Garrick, L. D., & Garrick, R. A. (1978). Temperature Influences on Hatchling *Caiman crocodilus* Distress Calls. *Physiological Zoology*, *51*(2), 105–113. https://doi.org/10.1086/physzool.51.2.30157859

Garrick, L. D., Lang, J. W., & Herzog, H. A. (1978). Social Signals of Adult American Alligators. *Bulletin of the American Museum of Natural History*, *160*, 155–192.

Herzog, H. A., & Burghardt, G. M. (1977). Vocalization in Juvenile Crocodilians. *Zeitschrift Für Tierpsychologie*, *44*(3), 294–304. https://doi.org/10.1111/j.1439-0310.1977.tb00997.x

Reber, S. A., Janisch, J., Torregrosa, K., Darlington, J., Vliet, K. A., & Fitch, W. T. (2017). Formants provide honest acoustic cues to body size in American alligators. *Scientific Reports*, *7*(1816), 1–11. https://doi.org/10.1038/s41598-017-01948-1

Reber, S. A., Nishimura, T., Janisch, J., Robertson, M., & Fitch, W. T. (2015). A Chinese alligator in heliox: Formant frequencies in a crocodilian. *J Exp Biol*, *218*, 2442–2447. https://doi.org/10.1242/jeb.119552

Riede, T., Tokuda, I. T., & Farmer, C. G. (2011). Subglottal pressure and fundamental frequency control in contact calls of juvenile *Alligator mississippiensis*. *Journal of Experimental Biology*, *214*(18), 3082–3095. https://doi.org/10.1242/jeb.051110

Roberto, I., & Botero-Arias, R. (2013). The distress call of *Caiman crocodilus crocodilus* (Crocodylia: Alligatoridae) in western Amazonia, Brazil. *Zootaxa*, *3647*, 593–596. https://doi.org/10.11646/zootaxa.3647.4.9

Sicuro, F. L., Iack-Ximenes, G. E., Wogel, H., & Bilate, M. (2013). Vocal patterns of adult females and juveniles Caiman yacare (Crocodilia: Alligatoridae) in Brazilian Pantanal wetland. *REVISTA DE BIOLOGIA TROPICAL*, *61*(3), 1401–1413.

Staniewicz, A., Foggett, S., McCabe, G., & Holderied, M. (2022). Courtship and underwater communication in the Sunda gharial (*Tomistoma schlegelii*). *Bioacoustics*, *31*(4), 435–449. https://doi.org/10.1080/09524622.2021.1967782

Staniewicz, A., McCabe, G., & Holderied, M. (2023). The low-frequency vocal repertoire of adult African dwarf crocodiles. *African Journal of Herpetology*, 1–16. https://doi.org/10.1080/21564574.2023.2237035

Todd, N. P. (2007). Estimated source intensity and active space of the American alligator (*Alligator Mississippiensis*) vocal display. *J Acoust Soc Am*, *122*(5), 2906–2915. https://doi.org/10.1121/1.2785811

Vergne, A. L., Aubin, T., Martin, S., & Mathevon, N. (2012). Acoustic communication in crocodilians: Information encoding and species specificity of juvenile calls. *Animal Cognition*, *15*(6), 1095–1109. https://doi.org/10.1007/s10071-012-0533-7

Vergne, A. L., Aubin, T., Taylor, P., & Mathevon, N. (2011). Acoustic signals of baby black caimans. *Zoology (Jena)*, *114*(6), 313–320. https://doi.org/10.1016/j.zool.2011.07.003

Vergne, A. L., Avril, A., Martin, S., & Mathevon, N. (2007). Parent-offspring communication in the Nile crocodile Crocodylus niloticus: Do newborns’ calls show an individual signature? *Die Naturwissenschaften*, *94*(1), 49–54. https://doi.org/10.1007/s00114-006-0156-4

Vergne, A. L., Pritz, M. B., & Mathevon, N. (2009). Acoustic communication in crocodilians: From behaviour to brain. *BIOLOGICAL REVIEWS*, *84*(3), 391–411. https://doi.org/10.1111/j.1469-185X.2009.00079.x

Vliet, K. A. (1989). Social Displays of the American Alligator (*Alligator mississippiensis*). *American Zoologist*, *29*(3), 1019–1031. https://doi.org/10.1093/icb/29.3.1019

Wang, X., Wang, D., Wu, X., Wang, R., & Wang, C. (2007). Acoustic signals of Chinese alligators (*Alligator sinensis*): Social communication. *The Journal of the Acoustical Society of America*, *121*(5), 2984–2989. https://doi.org/10.1121/1.2714910

**Table S5. Calculations**

Webb, G., & Messel, H. (1978). Morpohmetric Analysis of Crocodylus porosus from the North coast of Arnhem Land, Northern Australia. Australian Journal of Zoology, 26(1), 1-27. https://doi.org/10.1071/ZO9780001.

**Table S6. Glossary of terms**

Ajji M, J., & Lang, J. W. (2025). Gharial acoustic signaling: Novel underwater pops are temporally based, context-dependent, seasonally stable, male-specific, and individually distinctive. *Journal of Anatomy*. https://doi.org/10.1111/joa.14171

Beer, C. G. (1977). What Is a Display? *American Zoologist*, *17*(1), 155–165.

Bonke, R., Whitaker, N., Roedder, D., & Boehme, W. (2015). Vocalizations in two rare crocodilian species: A comparative analysis of distress calls of Tomistoma schlegelii (Müller, 1838) and Gavialis gangeticus (Gmelin, 1789). *North-Western Journal of Zoology*, *11*(1), 151–162.

Boucher, M., Tellez, M., & Anderson, J. T. (2020). Differences in distress: Variance and production of American Crocodile (*Crocodylus acutus*) distress calls in Belize. *Ecology and Evolution*, *10*(18), 9624–9634. https://doi.org/10.1002/ece3.6556

Chabert, T., Colin, A., Aubin, T., Shacks, V., Bourquin, S. L., Elsey, R. M., Acosta, J. G., & Mathevon, N. (2015). Size does matter: Crocodile mothers react more to the voice of smaller offspring. *Scientific Reports*, *5*(1), 15547–15547. <https://doi.org/10.1038/srep15547>

Charif, R.A., Strickman, L.M., & Waack, A.M. (2010). Raven Pro 1.4 User Manual. 11th ed. Cornell Lab of Ornithology, Ithaca NY, USA.

Erbe, C.A., Duncan, A., Hawkins, L., Terhune, J.M., & Thomas, J.A. (2022) Introduction to Acoustic Terminology and Signal Processing. 4. Springer Nature Switzerland AG & ASA Press. <https://doi.org/10.1007/978-3-030-97540-1>.

Garrick, L. D., & Garrick, R. A. (1978). Temperature Influences on Hatchling *Caiman crocodilus* Distress Calls. *Physiological Zoology*, *51*(2), 105–113. https://doi.org/10.1086/physzool.51.2.30157859

Garrick, L. D., Lang, J. W., & Herzog, H. A. (1978). Social Signals of Adult American Alligators. *Bulletin of the American Museum of Natural History*, *160*, 155–192.

Herzog, H. A., & Burghardt, G. M. (1977). Vocalization in Juvenile Crocodilians. *Zeitschrift Für Tierpsychologie*, *44*(3), 294–304. https://doi.org/10.1111/j.1439-0310.1977.tb00997.x

Odom, K. J., Araya-Salas, M., Morano, J. L., Ligon, R. A., Leighton, G. M., Taff, C. C., Dalziell, A. H., Billings, A. C., Germain, R. R., Pardo, M., de Andrade, L. G., Hedwig, D., Keen, S. C., Shiu, Y., Charif, R. A., Webster, M. S., & Rice, A. N. (2021). Comparative bioacoustics: A roadmap for quantifying and comparing animal sounds across diverse taxa. *BIOLOGICAL REVIEWS*, *96*(4), 1135–1159. https://doi.org/10.1111/brv.12695

Reber, S. A., Janisch, J., Torregrosa, K., Darlington, J., Vliet, K. A., & Fitch, W. T. (2017). Formants provide honest acoustic cues to body size in American alligators. *Scientific Reports*, *7*(1816), 1–11. https://doi.org/10.1038/s41598-017-01948-1

Reber, S. A., Nishimura, T., Janisch, J., Robertson, M., & Fitch, W. T. (2015). A Chinese alligator in heliox: Formant frequencies in a crocodilian. *J Exp Biol*, *218*, 2442–2447. https://doi.org/10.1242/jeb.119552

Riede, T., Tokuda, I. T., & Farmer, C. G. (2011). Subglottal pressure and fundamental frequency control in contact calls of juvenile *Alligator mississippiensis*. *Journal of Experimental Biology*, *214*(18), 3082–3095. https://doi.org/10.1242/jeb.051110

Roberto, I., & Botero-Arias, R. (2013). The distress call of *Caiman crocodilus crocodilus* (Crocodylia: Alligatoridae) in western Amazonia, Brazil. *Zootaxa*, *3647*, 593–596. https://doi.org/10.11646/zootaxa.3647.4.9

Sicuro, F. L., Iack-Ximenes, G. E., Wogel, H., & Bilate, M. (2013). Vocal patterns of adult females and juveniles Caiman yacare (Crocodilia: Alligatoridae) in Brazilian Pantanal wetland. *REVISTA DE BIOLOGIA TROPICAL*, *61*(3), 1401–1413.

Staniewicz, A., Foggett, S., McCabe, G., & Holderied, M. (2022). Courtship and underwater communication in the Sunda gharial (*Tomistoma schlegelii*). *Bioacoustics*, *31*(4), 435–449. https://doi.org/10.1080/09524622.2021.1967782

Staniewicz, A., McCabe, G., & Holderied, M. (2023). The low-frequency vocal repertoire of adult African dwarf crocodiles. *African Journal of Herpetology*, 1–16. https://doi.org/10.1080/21564574.2023.2237035

Todd, N. P. (2007). Estimated source intensity and active space of the American alligator (*Alligator Mississippiensis*) vocal display. *J Acoust Soc Am*, *122*(5), 2906–2915. https://doi.org/10.1121/1.2785811

Vergne, A. L., Aubin, T., Martin, S., & Mathevon, N. (2012). Acoustic communication in crocodilians: Information encoding and species specificity of juvenile calls. *Animal Cognition*, *15*(6), 1095–1109. https://doi.org/10.1007/s10071-012-0533-7

Vergne, A. L., Aubin, T., Taylor, P., & Mathevon, N. (2011). Acoustic signals of baby black caimans. *Zoology (Jena)*, *114*(6), 313–320. https://doi.org/10.1016/j.zool.2011.07.003

Vergne, A. L., Avril, A., Martin, S., & Mathevon, N. (2007). Parent-offspring communication in the Nile crocodile Crocodylus niloticus: Do newborns’ calls show an individual signature? *Die Naturwissenschaften*, *94*(1), 49–54. https://doi.org/10.1007/s00114-006-0156-4

Vergne, A. L., Pritz, M. B., & Mathevon, N. (2009). Acoustic communication in crocodilians: From behaviour to brain. *BIOLOGICAL REVIEWS*, *84*(3), 391–411. https://doi.org/10.1111/j.1469-185X.2009.00079.x

Vliet, K. A. (1989). Social Displays of the American Alligator (*Alligator mississippiensis*). *American Zoologist*, *29*(3), 1019–1031. https://doi.org/10.1093/icb/29.3.1019

Wildlife Acoustics (2024). Bandwidth. Glossary. Wildlife Acoustics MA, USA. <https://www.wildlifeacoustics.com/glossary/bandwidth>

Wang, X., Wang, D., Wu, X., Wang, R., & Wang, C. (2007). Acoustic signals of Chinese alligators (*Alligator sinensis*): Social communication. *The Journal of the Acoustical Society of America*, *121*(5), 2984–2989. https://doi.org/10.1121/1.2714910
